# Trem1 regulates neutrophil metabolism and recruitment in lung ischemia-reperfusion injury

**DOI:** 10.1016/j.redox.2026.104026

**Published:** 2026-01-14

**Authors:** Fengjing Yang, Song Tong, Junhao Wan, Yixing Li, Jiani Gao, Yan Sun, Xiangfu Sun, Huikang Fu, Wenzhuo Luo, Jiayang Xu, Ting Zhou, Sowe Babou, Junqi Wu, Guangjian Zhang, Chang Chen, Sihua Wang

**Affiliations:** a Department of Thoracic Surgery, Union Hospital, Tongji Medical College, Huazhong University of Science and Technology, Wuhan, 430022, China; b Department of Thoracic Surgery, The First Affiliated Hospital of Xi'an Jiaotong University, Xi'an, Shaanxi, 710061, China; c Department of Thoracic Surgery, Shanghai Pulmonary Hospital, Tongji University School of Medicine, Shanghai, 200433, China; d Department of Neurology, Union Hospital, Tongji Medical College, Huazhong University of Science and Technology, Wuhan, 430022, China; e Department of Critical Care Medicine, Union Hospital, Tongji Medical College, Huazhong University of Science and Technology, Wuhan, 430022, China

**Keywords:** Lung ischemia-reperfusion injury, Neutrophil, Trem1, Oxidative phosphorylation

## Abstract

Primary graft dysfunction (PGD) caused by ischemia-reperfusion injury (IRI) is a major complication after lung transplantation, yet its underlying mechanisms remain unclear. Triggering receptor expressed on myeloid cells 1 (Trem1) is an important mediator of inflammation, but its role in neutrophil function and metabolic reprogramming during lung IRI is not well understood. In this study, we used a murine orthotopic lung transplantation model with cold ischemia and reperfusion, and Trem1 knockout (*Trem1−/−*) and myeloid-specific Trem1 conditional knockout mice (*Lysm*^*Cre*^*Trem1*^*fl*^) to explore the role of Trem1 in neutrophil recruitment, neutrophil extracellular trap (NET) formation, and metabolism. Our results show that Trem1 expression increases in both mouse and human lungs after reperfusion and correlates with neutrophil infiltration and lung injury. Trem1 deficiency significantly reduced neutrophil and macrophage recruitment, NET formation, and tissue damage. Multi-omics analysis revealed that Trem1 deletion suppressed oxidative phosphorylation (OXPHOS) and induced a metabolic shift in neutrophils toward glycolysis. In clinical samples, the abundance of TREM1+ neutrophils was correlated with PGD severity and OXPHOS activity. These findings identify Trem1 as a key regulator of neutrophil metabolism and recruitment in lung IRI, and suggest that targeting Trem1 may provide a novel therapeutic strategy to mitigate PGD and improve lung transplant outcomes.

## Graphical abstract

**Figure:**
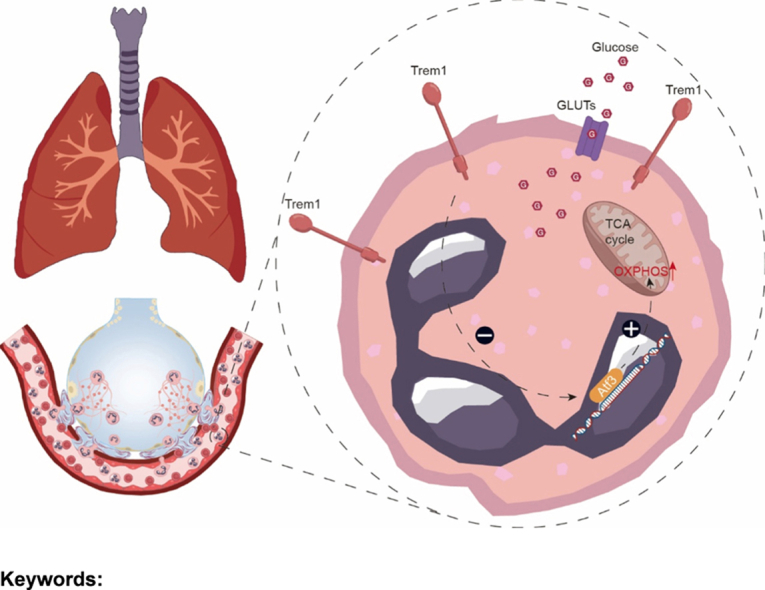

## 1 Introduction

Lung transplantation is the only effective treatment for patients with end-stage lung diseases, yet its success is limited by primary graft dysfunction (PGD), the leading cause of early morbidity and mortality after transplantation [1,2]. PGD is largely driven by ischemia-reperfusion injury (IRI), which triggers an intense inflammatory response characterized by neutrophil and macrophage infiltration, cytokine release, oxidative stress, and tissue damage [2]. Despite advances in perioperative care and immunosuppressive strategies, the mechanisms underlying PGD remain incompletely understood, and no effective targeted therapy is currently available.

Triggering receptor expressed on myeloid cells 1 (TREM1) is an immunoreceptor predominantly expressed on Myeloid-derived immune cells that amplifies inflammatory responses [3]. TREM1 has been implicated in sepsis, acute lung injury, and transplant rejection, but its role in lung transplantation-associated IRI and PGD has not been fully defined. In particular, emerging evidence suggests that immune cell metabolism is critical for shaping cellular recruitment and effector functions [[4], [5], [6]]. However, whether TREM1 regulates neutrophil recruitment and function through metabolic reprogramming in the context of lung IRI remains unknown.

Recent studies have highlighted the importance of metabolic regulation in shaping the function of myeloid immune cells [7]. Macrophage metabolism has been extensively investigated, with distinct reliance on glycolysis or oxidative phosphorylation depending on their activation states [8,9]. In contrast, much less is known about neutrophil metabolism, particularly their energy metabolism, in the setting of transplantation. Although neutrophils are classically considered short-lived glycolytic cells, emerging evidence suggests that mitochondrial activity and oxidative phosphorylation (OXPHOS) may play critical roles in sustaining their recruitment, effector functions, and NET formation [[10], [11], [12]]. However, how neutrophil metabolic programs are regulated in lung IRI remains poorly understood.

Activating transcription factor 3 (ATF3), a member of the ATF/CREB family, is an immediate early gene induced by cellular stress and inflammatory stimuli [13,14]. ATF3 has been reported to act as a critical regulator of innate immune responses, modulating Toll-like receptor signaling and shaping macrophage polarization [15,16]. However, its role in neutrophils, particularly in controlling energy metabolism and neutrophil extracellular trap (NET) formation, remains poorly defined. Whether ATF3 participates in the metabolic reprogramming of neutrophils during ischemia-reperfusion injury and contributes to the development of primary graft dysfunction is unknown.

Here, we investigated the role of TREM1 in experimental lung transplantation using global Trem1 knockout mice, myeloid-specific Trem1 conditional knockout mice, and multi-omics approaches including bulk RNA-seq, single-cell RNA-seq, ATAC-seq, and metabolic profiling. We further validated our findings in human lung transplant samples stratified by PGD severity. Our study reveals a previously unrecognized mechanism in which TREM1 promotes neutrophil recruitment and NET formation by modulating OXPHOS through Atf3, thereby exacerbating lung IRI and PGD. These findings identify the TREM1–Atf3–OXPHOS axis as a potential therapeutic target for mitigating PGD in lung transplantation.

## 2 Materials and methods

### 2.1 Mice

All animal experiments were reviewed and approved by the Animal Care and Use Committee of Tongji Medical College, Huazhong University of Science and Technology (approval no. S4817), and were performed in accordance with institutional and national guidelines for the care and use of laboratory animals. Specific pathogen–free (SPF) male C57BL/6J mice (6–8 weeks old, 22–28 g) were obtained from Shulaibao (Wuhan) Biotechnology Co., Ltd. and used as both donors and recipients. Trem1-deficient (*Trem1−/−*) mice on a C57BL/6J background were generated by backcrossing for at least six generations and validated by PCR genotyping to confirm the absence of functional Trem1 alleles. Conditional myeloid-specific Trem1 knockout mice (*Lysm*^*Cre*^*Trem1*^*fl*^) were established by crossing *Lysm*^*Cre*^ transgenic mice with *Trem1*^*fl*^ mice, with littermate *Trem1*^*fl*^ mice serving as controls. All animals were maintained under controlled conditions (22 ± 2 °C, 50 %–60 % relative humidity, 12-h light/dark cycle) with ad libitum access to food and water. All procedures were conducted with efforts to minimize animal suffering.

### 2.2 Human lung samples

Human lung tissue specimens were obtained from lung transplant recipients at the First Affiliated Hospital of Xi'an Jiaotong University (Xi'an, China) between 2022 and 2023. A total of 25 patients were enrolled, including donor lungs subjected to cold ischemia (CIT, *n* = 5) and graft tissues collected after reperfusion from recipients stratified by ISHLT primary graft dysfunction (PGD) grades 0–3 (*n* = 5 per group). Clinical information and PGD grading were determined according to ISHLT consensus criteria.

All tissue procurement followed the Chinese Clinical Guidelines for Lung Transplant Biobank Construction (2025 edition), with standardized procedures for sample collection, cold ischemia control, and preservation to ensure tissue integrity and reproducibility. Samples were processed within 30 min of retrieval whenever possible, and stored either in formalin for histological evaluation or in cryopreservation medium for subsequent analyses.

For histological evaluation, paraffin-embedded lung sections (4 μm) were prepared for hematoxylin–eosin (HE) and immunofluorescence staining to assess tissue injury, neutrophil infiltration, and TREM1 expression. For flow cytometry, fresh lung tissues were enzymatically digested into single-cell suspensions, followed by red blood cell lysis and antibody staining as described above. Neutrophils were further purified using magnetic-activated cell sorting (MACS), and purity (>90 %) was confirmed by flow cytometry. Purified neutrophils were subjected to Seahorse XF analysis to measure oxygen consumption rate (OCR) and extracellular acidification rate (ECAR).

The study protocol was reviewed and approved by the Institutional Review Board (IRB) of the First Affiliated Hospital of Xi'an Jiaotong University (Approval No. XJTU1AFCRC2023SJ-006). Written informed consent was obtained from all participants or their legal representatives prior to sample collection, in accordance with the Declaration of Helsinki and Chinese regulations on biosafety and human genetic resource management.

### 2.3 Murine orthotopic lung transplantation and reperfusion model

A murine orthotopic left lung transplantation model was established using a modified cuff technique, as previously described [17](ref). Briefly, donor C57BL/6J or gene-targeted *Trem1−/−* mice were anesthetized and intubated. The left lung was harvested under sterile conditions, and the pulmonary artery, pulmonary vein, and main bronchus were dissected free. An 18G, 22G, and 24G cuff were placed on the bronchus, pulmonary vein, and pulmonary artery, respectively, and secured with 9-0 sutures. The graft was flushed with cold (4 °C) low-potassium dextran (Perfadex®) solution and stored on ice for the indicated ischemic duration, with the lung kept inflated and the bronchus clamped to prevent fluid entry.

Recipient mice were anesthetized, intubated, and mechanically ventilated, followed by a left thoracotomy through the third intercostal space. The left pulmonary hilum was carefully dissected, and vascular clamps were applied to the pulmonary artery and vein. Sequential anastomoses of the donor pulmonary vein, pulmonary artery, and bronchus were performed using the cuff technique, after which vascular flow was restored. Following confirmation of adequate graft ventilation, the native left lung was removed, the graft was placed in the thoracic cavity, and the thoracotomy was closed. Grafts were harvested 4 h after reperfusion for histological, molecular, and cellular analyses.

For the cold ischemia (CIT) control group, donor lungs were subjected to the same duration of cold ischemia followed by normothermic incubation at 37 °C for an equivalent period, but without vascular anastomosis or reperfusion.

### 2.4 Histology and immunofluorescence

Lung grafts were harvested 4 h after reperfusion, fixed in 4 % paraformaldehyde for 24 h, embedded in paraffin, and sectioned at 4 μm thickness. Hematoxylin and eosin (H&E) staining was performed to evaluate structural injury, inflammatory cell infiltration, interstitial edema, and hemorrhage.

For immunofluorescence staining, sections were deparaffinized, rehydrated, and subjected to antigen retrieval. Primary antibodies were applied to detect immune cell subsets and functional markers, including Trem1 (clone EPR26206-72, Abcam, ab300461), Ly6G (Servicebio, GB12229-100), F4/80 (Servicebio, GB12027-100), MPO (Abcam, ab208670), citrullinated histone H3 (CitH3; [RM1001], Abcam, ab281584), CD66B (Sino Biological, S-766-55), JUN (c-Jun Recombinant Superclonal™, 2HCLC; Thermo Fisher), and ATF3 (clone CL1685, Thermo Fisher). Alexa Fluor–conjugated secondary antibodies (488, 594, 647; Absin) were used for detection.

Stained sections were imaged with a Zeiss LSM 880 confocal microscope. Quantitative analysis was performed using ImageJ software, with five randomly selected high-power fields per section analyzed for positive cell counts. Marker expression was reported as both percentage of positive cells and positive area fraction.

### 2.5 Flow cytometry

Lung grafts were harvested 4 h after reperfusion, perfused with PBS to remove residual blood, minced, and digested to generate single-cell suspensions, which were filtered through a 70-μm strainer. Red blood cells were lysed with RBC lysis buffer. To block nonspecific Fc receptor binding, cells were incubated with purified anti-mouse CD16/CD32. Cells were then stained with fluorochrome-conjugated antibodies for immune cell subset identification.

Human lung grafts were processed within 1 h of retrieval. Tissues were perfused with PBS, enzymatically digested, filtered through a 70-μm strainer, and subjected to RBC lysis. After Fc receptor blocking (Human TruStain FcX™, BioLegend, 422301), cells were stained with fluorochrome-conjugated antibodies.

### 2.6 Seahorse metabolic flux analysis

Neutrophils were isolated by magnetic bead sorting (anti-Ly6G, Miltenyi Biotec) and seeded into Seahorse XF96 cell culture microplates (Agilent Technologies). Cellular bioenergetics were assessed using the Seahorse XF Cell Mito Stress Test Kit and the Seahorse XF Glycolytic Rate Assay Kit (Agilent Technologies), following the manufacturer's instructions. Oxygen consumption rate (OCR) and extracellular acidification rate (ECAR) were measured in real time with a Seahorse XF Analyzer. Sequential injections of oligomycin (1 μM), FCCP (1 μM), and rotenone/antimycin A (0.5 μM each) were used to determine basal respiration, ATP production, maximal respiratory capacity, and spare respiratory capacity. Glycolytic parameters, including basal glycolysis and compensatory glycolysis, were quantified from ECAR measurements. Data were analyzed using Wave software (Agilent Technologies).

### 2.7 Neutrophil isolation by magnetic bead sorting

Neutrophils were isolated from murine graft tissues using the Neutrophil Isolation Kit, mouse (Miltenyi Biotec, 130-097-658) in combination with LS Separation Columns (Miltenyi Biotec, 130-042-401), according to the manufacturer's instructions. Briefly, grafted left lungs were enzymatically digested to obtain single-cell suspensions, followed by red blood cell lysis (Solarbio, R1010). Ly6G MicroBeads (Miltenyi Biotec) were then used for magnetic labeling and enrichment of neutrophils. The purity of isolated neutrophils was routinely verified by flow cytometry, and only preparations with ≥90 % purity were used (Fig. S6C). Purified neutrophils were subjected to downstream applications, including RNA extraction, metabolic flow cytometry, and Seahorse assays.

### 2.8 Bioinformatics analyses

Bulk RNA-seq. Raw sequencing reads were subjected to quality control with *FastQC*, and low-quality reads and adapter sequences were trimmed using *Trimmomatic*. Clean reads were aligned to the mouse reference genome (mm10) using *HISAT2* or *STAR*. Gene-level read counts were obtained, and differential expression analysis was performed with *DESeq2*, applying thresholds of adjusted P < 0.05 and |log2 fold change| > 1. Functional enrichment analyses of differentially expressed genes were conducted with the *clusterProfiler* package, focusing on Gene Ontology (GO) terms and KEGG pathways.

Single-cell RNA-seq (scRNA-seq). Raw scRNA-seq data were processed with *Cell Ranger* for quality control, alignment, and quantification. Downstream analysis was performed with *Seurat*, including normalization, clustering, dimensionality reduction (UMAP/t-SNE), and identification of marker genes. Subcluster analyses were carried out using *SeuratWrappers*. Differentially expressed genes were compared against canonical immune cell markers for annotation. Functional enrichment was performed with *GSVA* and *clusterProfiler* to identify pathway-level differences across immune subsets.

ATAC-seq. ATAC-seq reads were quality controlled using *FastQC* and trimmed with *Trimmomatic*. Reads were aligned to the mm10 genome using *Bowtie2*, and peaks were called with *MACS2* to define accessible chromatin regions. *Homer* was used for motif enrichment analysis to identify putative transcription factor binding sites. Integration of ATAC-seq and RNA-seq data was performed to evaluate the relationship between chromatin accessibility and transcriptional regulation in metabolic and immune pathways.

Visualization and statistics. All bioinformatics analyses were performed in *R* (v4.1) and Linux environments. Visualization was carried out using *ggplot2*, *ComplexHeatmap*, and *plotly*. Pathway enrichment analyses were performed with *clusterProfiler* and *ReactomePA*. Statistical comparisons were performed using Student's *t*-test or ANOVA, and P < 0.05 was considered statistically significant.

### 2.9 Western blotting

Lung grafts were harvested 4 h after reperfusion, and total protein was extracted using RIPA lysis buffer supplemented with protease inhibitors. Protein concentrations were quantified with a BCA protein assay kit (Beyotime, P0012). Equal amounts of protein (20–30 μg) were separated by SDS–PAGE and transferred onto PVDF membranes. Membranes were blocked with 5 % nonfat milk in TBST and incubated overnight at 4 °C with primary antibody against Trem1 (Abcam, ab104413; 1:1000). After washing, membranes were incubated with HRP-conjugated secondary antibody and developed with enhanced chemiluminescence (ECL, Bio-Rad). Protein bands were visualized using the ChemiDoc Imaging System (Bio-Rad), and densitometry was performed with ImageJ software.

### 2.10 Quantitative Real-Time PCR (qPCR)

Lung grafts were harvested 4 h after reperfusion, and total RNA was extracted using TRIzol reagent (Invitrogen). cDNA was synthesized with the ABScript III RT Master Mix (ABclonal, Wuhan, China) according to the manufacturer's protocol. qPCR was performed using SYBR Green Master Mix (ABclonal) on a StepOnePlus Real-Time PCR System (Applied Biosystems). The primers for Trem1 were: forward 5′-GCCTTGTGCCCACTCTATACCA-3′, reverse 5′-TGGAGACATCGGCAGTTGAC-3′. β-actin was used as an internal control (forward 5′-GTGGCCGAGGACTTTGATTG-3′, reverse 5′-CCTGTAACAACGCATCTCATATT-3′). Relative Trem1 expression levels were calculated using the 2ˆ−ΔΔCt method.

### 2.11 Transmission electron microscopy

Purified neutrophils were fixed in 2.5 % glutaraldehyde (Electron Microscopy Sciences) at 4 °C overnight. Following fixation, cells were washed in phosphate-buffered saline (PBS), post-fixed in 1 % osmium tetroxide, and dehydrated through a graded ethanol series. Samples were embedded in epoxy resin and ultrathin sections (70 nm) were prepared using an ultramicrotome. Sections were stained with uranyl acetate and lead citrate and examined with a JEM-1400Plus transmission electron microscope (JEOL). High-resolution images were acquired to assess subcellular ultrastructure, including mitochondria and endoplasmic reticulum. Morphometric quantification of mitochondrial morphology and number was performed using ImageJ software.

### 2.12 ELISA assay

Cytokine concentrations in bronchoalveolar lavage fluid (BALF) and serum were determined using commercially available ELISA kits. The assays were performed according to the manufacturer's protocols (Shanghai Jianglai Biotechnology Co., Ltd). Briefly, samples or standards were added to wells pre-coated with capture antibodies, followed by incubation with biotinylated detection antibodies and horseradish peroxidase (HRP)-conjugated secondary antibodies. The substrate 3,3′,5,5′-tetramethylbenzidine (TMB) was then added for colorimetric detection. The reaction was terminated under acidic conditions, and absorbance was measured at 450 nm using a microplate reader (BioTek Epoch). Standard curves were generated from known concentrations, and cytokine levels were quantified based on the corresponding OD values. The following ELISA kits were used: mouse IL-1β (Cat# JL18442), TNF-α (Cat# JL10484), and IL-6 (Cat# JL20268).

## 3 Results

### 3.1 Trem1 is upregulated in murine and human lungs following ischemia-reperfusion

**Fig. 1:**
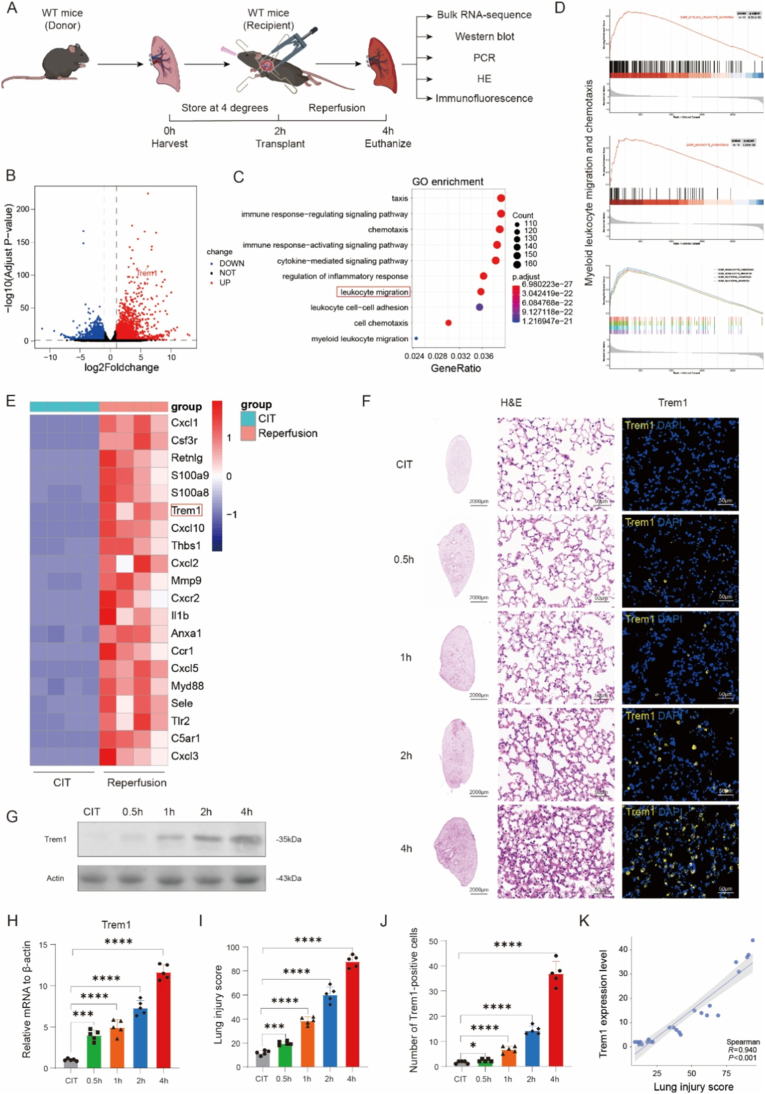
**Trem1 expression is increased in a murine lung transplantation model with cold ischemia and reperfusion injury.** (A) Experimental schematic: wild-type (WT) donor lungs were subjected to 2 h of cold ischemia (CIT) prior to orthotopic transplantation into WT recipients, followed by 2–4 h of reperfusion. For the CIT control group, donor lungs were subjected to 2 h of cold ischemia followed by 2 h of normothermic incubation at 37 °C without vascular reperfusion.Grafts were analyzed by bulk RNA-seq (n = 4/group), histology, immunofluorescence, qPCR, and Western blot (n = 5/group). (B) Volcano plot showing differentially expressed genes between CIT and reperfusion groups, with Trem1 significantly upregulated. (C) Gene Ontology enrichment of differentially expressed genes, highlighting leukocyte migration and inflammatory signaling pathways. (D) Gene set enrichment analysis (GSEA) demonstrating enrichment of chemotaxis- and myeloid migration–related signatures. (E) Heatmap of representative leukocyte migration–associated genes, including Trem1. (F) Representative H&E staining and Trem1 immunofluorescence in grafts at different reperfusion time points. Scale bars: 2000 μm (whole lung), 50 μm (high magnification). (G–H) Western blot and qPCR analysis showing progressive upregulation of Trem1 with reperfusion. (I) Quantification of lung injury scores at indicated reperfusion times. (J) Quantification of Trem1^+^ cells by immunofluorescence. (K) Correlation between Trem1 expression levels and lung injury scores, demonstrating a strong positive association. Data are shown as mean ± SEM (n = 4–5/group). Statistical significance was determined by one-way ANOVA with post hoc testing. ∗P < 0.05, ∗∗P < 0.01, ∗∗∗P < 0.001, ∗∗∗∗P < 0.0001.

To identify key determinants of primary graft dysfunction (PGD), we employed a murine orthotopic lung transplantation model that closely mimics the clinical setting [18]. Bulk RNA sequencing was performed on wild-type donor lungs after 2 h of cold ischemia (CIT) and after 2 h of reperfusion following transplantation (Reperfusion) (Fig. 1A), Importantly, to control for temperature-dependent transcriptional changes, lungs in the CIT group were subjected to 2 h of cold ischemia followed by 2 h of normothermic incubation at 37 °C, but without vascular reperfusion.Differential expression analysis (|log2 fold change| > 1, *P* < 0.05) identified 6408 genes, including 3147 upregulated and 3261 downregulated genes (Fig. 1B). Gene Ontology (GO) enrichment revealed significant enrichment in pathways related to leukocyte migration, inflammatory regulation, chemokine-mediated signaling, and immune activation, with leukocyte migration emerging as the most significant pathway (Fig. 1C). Gene set enrichment analysis (GSEA) further demonstrated strong enrichment of myeloid cell, particularly neutrophil, chemotaxis and migration signatures (Fig. 1D).

Among the top 20 differentially expressed genes associated with leukocyte migration (Fig. 1E), Trem1 stood out given its high expression in neutrophils, monocytes, and tissue macrophages, and its well-established role in amplifying inflammatory responses [19]. Notably, Trem1 expression was increased in transplanted lungs at 2 h post-reperfusion [20] (Fig. S1B) and further elevated with prolonged reperfusion [21] (Fig. S1A). Consistent with these findings, in our 4-h reperfusion model, Trem1 mRNA and protein levels progressively increased with reperfusion time (Fig. 1F–H, J) and correlated positively with histological lung injury scores (Fig. 1I–K).

To assess clinical relevance, we analyzed transcriptomic datasets from human lung allografts following reperfusion. Stratification by TREM1 expression revealed significant differences in neutrophil abundance between high- and low-TREM1 groups as determined by CIBERSORT (Fig. S1C), with neutrophil infiltration positively correlating with TREM1 expression (Fig. S1D).

These findings establish Trem1 as an early and dynamic marker of lung ischemia-reperfusion injury, with expression most prominently elevated at 4 h of reperfusion. This observation not only validates the use of the 2-h cold ischemia/4-h reperfusion transplantation model [22], but also provides a mechanistic rationale to further investigate Trem1 as a driver of neutrophil recruitment and graft injury.

### 3.2 Trem1 deficiency attenuates lung ischemia-reperfusion injury after transplantation in mice

**Fig. 2:**
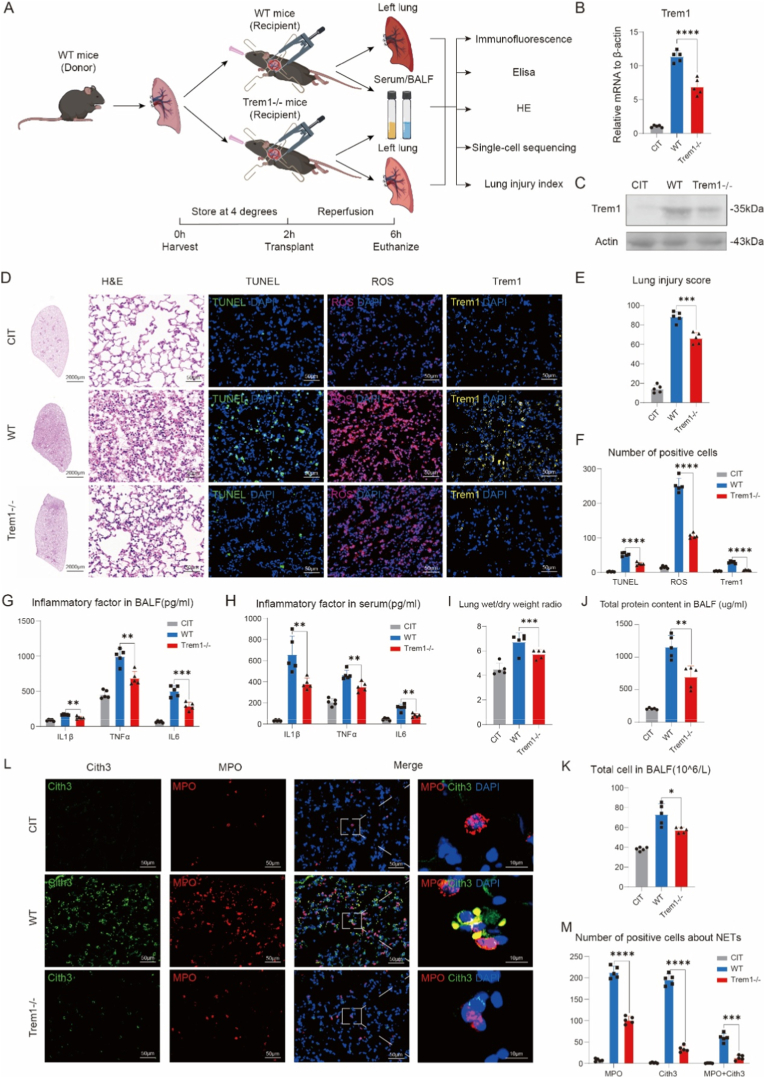
**Trem1 deficiency attenuates acute lung injury following transplantation and reperfusion.** (A) Schematic of orthotopic lung transplantation with 2 h cold ischemia (CIT) and 4 h reperfusion, with collection of graft tissue, bronchoalveolar lavage fluid (BALF), and serum for analysis (n = 5/group). For the CIT control group, donor lungs were subjected to 2 h of cold ischemia followed by normothermic incubation at 37 °C for an equivalent duration, but without vascular reperfusion. (B–C) Trem1 expression in grafts assessed by qPCR (B) and Western blot (C). (D) Representative H&E staining, TUNEL, ROS, and Trem1 immunofluorescence in graft tissue. Scale bars: 2000 μm (H&E low magnification), 50 μm (high magnification). (E) Quantification of lung injury scores. (F) Quantification of TUNEL+, ROS+, and Trem1+ cells by immunofluorescence. (G–H) Concentrations of IL-1β, TNF-α, and IL-6 in BALF (G) and serum (H). (I–K) Lung wet-to-dry weight ratio (I), BALF protein content (J), and total BALF cell counts determined by flow cytometric analysis (K). (L) Representative immunofluorescence of NETs, as indicated by CitH3 and MPO co-staining. Scale bars: 50 μm (overview), 10 μm (zoom). (M) Quantification of CitH3^+^ and MPO^+^ cells in lung graft tissue. NET formation was defined by the presence of MPO^+^CitH3^+^ double-positive cells, while CitH3-only and MPO-only single-positive cells were quantified separately. Positive cells were quantified from five randomly selected high-power fields per section using ImageJ. Data are presented as mean ± SEM (n = 5/group). Statistical significance was determined by one-way ANOVA with post hoc correction. ∗P < 0.05, ∗∗P < 0.01, ∗∗∗P < 0.001, ∗∗∗∗P < 0.0001.

To investigate the functional consequences of Trem1 in lung ischemia-reperfusion injury, we utilized global Trem1 knockout mice (*Trem1−/−*) (Fig. S2). Donor lungs from wild-type mice were subjected to 2 h of cold ischemia at 4 °C and transplanted into either wild-type or *Trem1−/−* recipients, followed by 4 h of reperfusion (Fig. 2A). Lungs in the CIT control group underwent the same duration of cold ischemia followed by normothermic incubation at 37 °C for an equivalent period, but without vascular reperfusion. As expected, Trem1 expression was markedly reduced in grafts from *Trem1−/−* recipients compared with wild-type controls (Fig. 2B and C).

Histological analysis revealed that Trem1 deficiency significantly alleviated lung injury, as evidenced by reduced alveolar damage, inflammatory cell infiltration, and apoptosis, together with diminished ROS generation (Fig. 2D–F). Consistently, Trem1−/− recipients exhibited lower levels of TNF-α, IL-1β, and IL-6 in bronchoalveolar lavage fluid (BALF) and serum, as well as reduced cellularity, protein leakage, and wet-to-dry ratios in the transplanted lungs (Fig. 2G–K).In contrast, transplantation of lungs from *Trem1−/−* or *Lysm*^*Cre*^*Trem1*^*fl*^ donors into wild-type recipients did not result in a significant attenuation of lung injury or inflammatory responses (Figs. S3 and S4).

Given the strong association between neutrophil extracellular traps (NETs) and primary graft dysfunction after lung transplantation [23], and the established role of Trem1 in NET formation [6,24], we further examined MPO and citrullinated histone H3 (CitH3) as markers of NETs. Notably, Trem1 deficiency significantly suppressed NET formation in reperfused lung grafts (Fig. 2L and M).

Together, these findings demonstrate that Trem1 plays a critical role in mediating lung ischemia-reperfusion injury and NET formation, and that its deletion confers protection against acute graft injury.

### 3.3 Trem1 deficiency reduces myeloid cell recruitment, particularly neutrophils, during lung reperfusion

Innate and adaptive immune cells, including T cells, B cells, NK cells, neutrophils, and macrophages, contribute to the development of primary graft dysfunction following lung transplantation [[25], [26], [27], [28], [29], [30], [31]]. Given the protective effects of Trem1 deletion against ischemia-reperfusion injury (Fig. 2), we next investigated which immune cell populations were primarily affected by Trem1 deficiency.

**Fig. 3:**
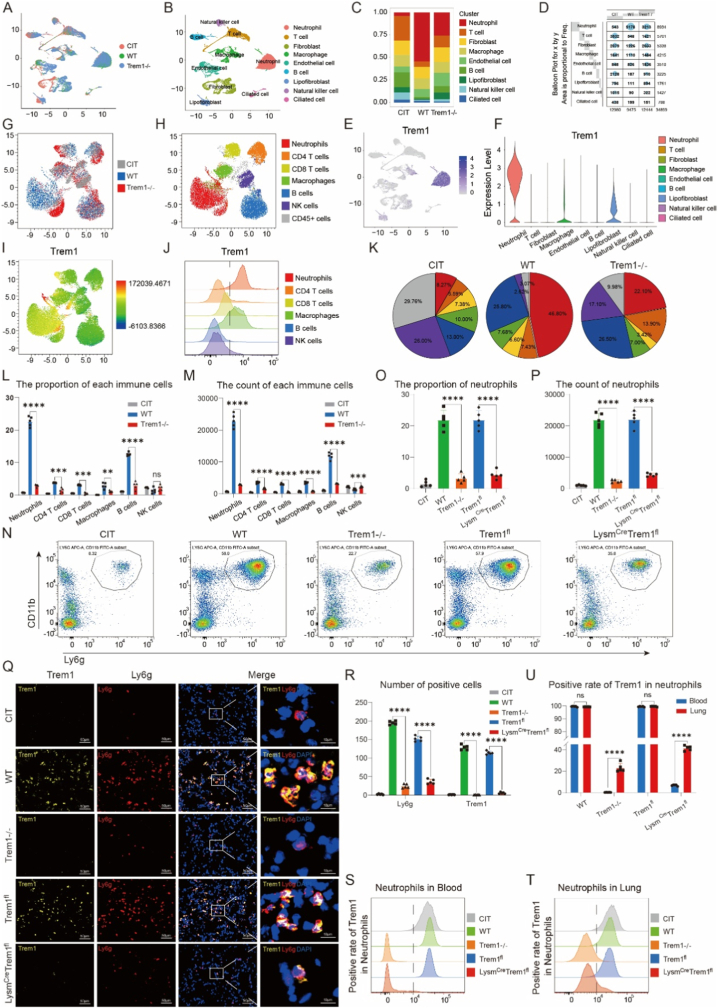
**Trem1 deficiency reduces neutrophil recruitment during lung transplantation–induced reperfusion injury.** (A–B) UMAP visualization of scRNA-Seq data from graft tissue, showing clustering of cells by sample group (A) and by cell type (B). (C–D) Proportional abundance (C) and absolute counts (D) of each cell cluster across groups. (E–F) UMAP and violin plots showing Trem1 expression across immune and stromal populations. (G–H) Flow cytometry–based UMAP plots of CD45^+^ immune subsets from grafts. (I–J) Distribution and density of Trem1 expression across neutrophils, macrophages, T cells, B cells, and NK cells. (K–M) Relative proportions (K, L) and absolute counts (M) of immune cell subsets in CIT, WT, and *Trem1−/−* recipients. (N–P) Flow cytometry quantification of neutrophil proportions (O) and total numbers (P) per 10^6^ graft cells in CIT, WT, *Trem1−/−*, *Trem1*^*fl*^, and *Lysm*^*Cre*^*Trem1*^*fl*^ mice. (Q–R) Representative immunofluorescence images (Q) and quantification (R) of Trem1^+^Ly6G^+^ neutrophils in grafts. Scale bars: 50 μm (overview), 10 μm (zoom). (S–T) Representative histograms of Trem1 positivity in neutrophils from blood (S) and lung grafts (T). (U) Quantification of Trem1^+^ neutrophils in blood versus lung grafts across genotypes. For scRNA-Seq, one sample per group was analyzed; for flow cytometry and immunofluorescence validation, n = 5/group. Data are presented as mean ± SEM. Statistical significance was determined by one-way ANOVA with post hoc correction. ∗P < 0.05, ∗∗P < 0.01, ∗∗∗P < 0.001, ∗∗∗∗P < 0.0001.

Single-cell RNA sequencing (scRNA-seq) was performed on donor lungs subjected to cold ischemia followed by normothermic incubation, as well as on grafts harvested from wild-type and *Trem1−/−* recipients after 4 h of reperfusion (Fig. 3). Clustering analysis identified nine major cell populations, including neutrophils, T cells, fibroblasts, macrophages, endothelial cells, B cells, lipofibroblasts, NK cells, and ciliated epithelial cells (Fig. 3A and B; Fig. S5). This clustering pattern was consistent with a recently published lung transplant dataset (GSE237110) (Fig. S6A and B).

Among these populations, neutrophils exhibited the most striking changes, both in absolute numbers and proportions. *Trem1−/−* recipients showed a marked reduction in neutrophil recruitment compared with wild-type recipients after reperfusion (Fig. 3C and D). This conclusion was further supported by flow cytometry validation (Fig. 3K–M) and confirmed using GSE237110 (Fig. S6C and D). In contrast, transplantation of lungs from *Trem1−/−* or *Lysm*^*Cre*^*Trem1*^*fl*^ donors into wild-type recipients did not result in a significant attenuation of lung injury, inflammatory cytokine production, or neutrophil accumulation following reperfusion (Figs. S3 and S4). These findings suggest that Trem1 expression in recipient-derived immune cells, rather than in donor lung parenchyma alone, is critical for mediating lung ischemia–reperfusion injury.

To exclude indirect effects on other immune cells, we generated myeloid-specific Trem1 conditional knockout mice (*Lysm*^*Cre*^*Trem1*^*fl*^) (Fig. S8A). Both global Trem1 knockout and myeloid-specific Trem1 deletion significantly reduced neutrophil recruitment, as demonstrated by flow cytometry and immunofluorescence (Fig. 3N–R). In addition to neutrophils, Trem1 deficiency also attenuated macrophage recruitment, a finding validated in both global and conditional knockout mice (Fig. S9).

Together, these results demonstrate that Trem1 deficiency primarily impairs the recruitment of myeloid-derived immune cells during lung reperfusion, with neutrophils representing the most affected population.

### 3.4 Trem1 exhibits distinct expression dynamics in neutrophils and macrophages during lung reperfusion

We next examined the cellular distribution of Trem1 expression across lung immune populations. scRNA-seq revealed that Trem1 was predominantly expressed in myeloid-derived cells, including neutrophils, macrophages, and lipofibroblasts (Fig. 3E and F). This finding was validated by flow cytometry (Fig. 3G–J) and confirmed in an independent single-cell dataset of lung transplantation [32] (Fig. S6E and F), consistent with previous reports [5]. Importantly, compositional analysis of Trem1-positive cells by flow cytometry demonstrated that neutrophils constituted the predominant immune cell subset within the Trem1^+^ population in lung tissue (Fig. S7).

Interestingly, Trem1 displayed divergent expression patterns between neutrophils and macrophages. In *Trem1−/−* mice, the frequencies of neutrophils and macrophages in blood, spleen, and bone marrow were comparable to wild-type controls (Figs. S2E, F, H, I), indicating that reduced myeloid recruitment after transplantation was not due to baseline differences in cell numbers. However, Trem1 positivity differed by cell type. In neutrophils, Trem1 expression was consistently high in wild-type mice across blood, spleen, and bone marrow, whereas *Trem1−/−* mice showed markedly reduced Trem1 positivity. Notably, bone marrow neutrophils exhibited lower Trem1 positivity compared with blood and splenic neutrophils, and flow cytometry revealed a distinct subpopulation pattern in wild-type bone marrow neutrophils (Fig. S2D and G).

By contrast, macrophages expressed Trem1 at low baseline levels (<10 %) across all compartments, regardless of Trem1 status (Fig. S2J). Following lung transplantation, however, macrophage Trem1 positivity markedly increased in wild-type and *Trem1*^*fl*^ mice, with a less pronounced induction observed in *Trem1−/−* and *Lysm*^*Cre*^*Trem1*^*fl*^ mice (Fig. S9H–J). These findings suggest that macrophage Trem1 expression is inducible and dependent on transplantation-associated inflammatory cues.

Consistent with this, comparison of circulating neutrophils at baseline and graft-infiltrating neutrophils post-transplant revealed uniformly high Trem1 expression (>95 %) in wild-type and *Trem1*^*fl*^ mice. In *Trem1−/−* and *Lysm*^*Cre*^*Trem1*^*fl*^ mice, neutrophil Trem1 positivity was markedly reduced in circulation but partially restored upon infiltration into the transplanted lung (Fig. 3S–U). In contrast, macrophages exhibited inducible Trem1 expression in all genotypes upon reperfusion, albeit at different magnitudes.

Together, these results reveal a cell type–specific pattern of Trem1 regulation: Trem1 is constitutively expressed in neutrophils and partially restored upon stimulation even in Trem1-deficient mice, whereas macrophages exhibit inducible Trem1 expression triggered by transplantation injury.

### 3.5 Trem1 reprograms energy metabolism in myeloid cells during lung reperfusion

**Fig. 4:**
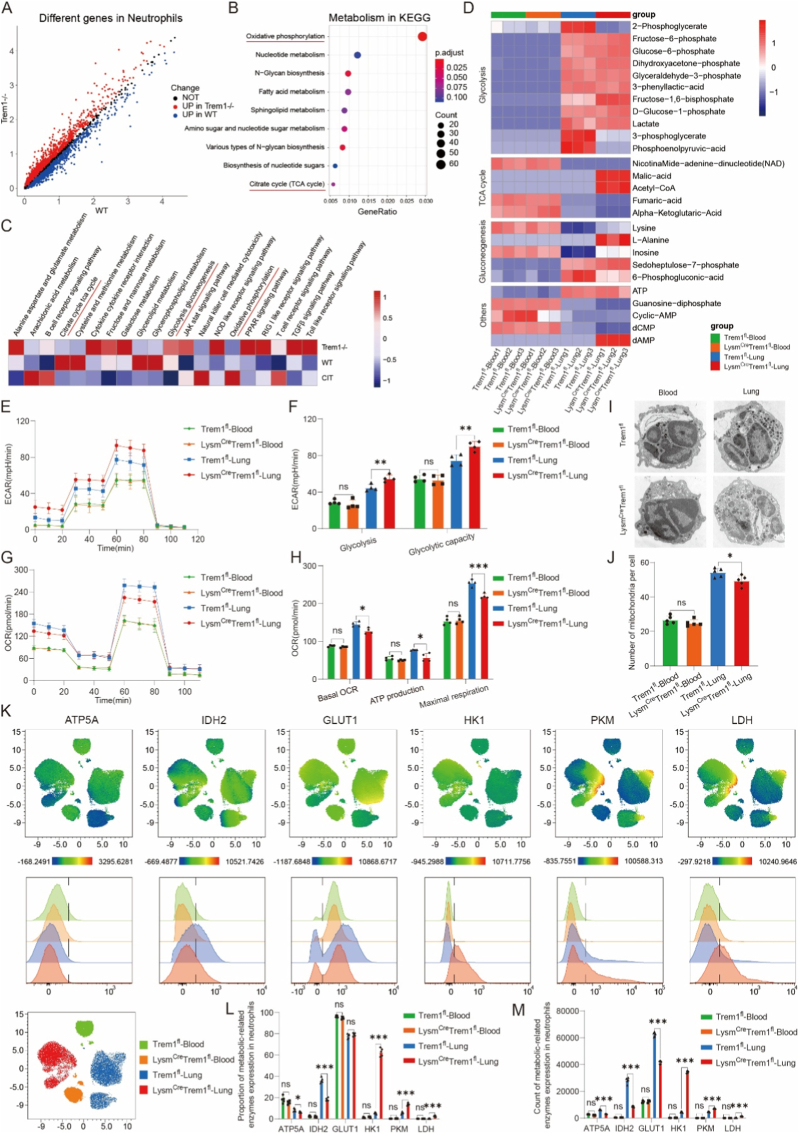
**Trem1 deletion suppresses oxidative phosphorylation in activated neutrophils.** (A) Gene-wise expression comparison of graft-infiltrating neutrophils identified by single-cell RNA sequencing. Each dot represents a single gene. The x-axis denotes the average expression level of each gene in neutrophils from wild-type grafts, while the y-axis denotes the corresponding expression level in neutrophils from *Trem1−/−* grafts. Neutrophils were computationally defined based on canonical marker genes and were not physically isolated by flow cytometry for this analysis. (B) KEGG pathway enrichment analysis of differentially expressed genes within neutrophil clusters derived from scRNA-seq data. (C) Heatmap showing gene set enrichment analysis (GSEA) scores of metabolic pathways across individual neutrophils. (D) Targeted metabolomics profiling of glycolysis, TCA cycle, and gluconeogenesis intermediates in neutrophils from blood and graft tissue. (E–F) Seahorse extracellular flux analysis showing extracellular acidification rate (ECAR) (E) and glycolytic flux (F) in blood- and graft-derived neutrophils from *Trem1*^*fl*^ and *Lysm*^*Cre*^*Trem1*^*fl*^ mice. (G–H) Oxygen consumption rate (OCR) traces (G) and quantification of basal respiration, ATP production, and maximal respiration (H). (I–J) Transmission electron microscopy of neutrophils (I) with quantification of mitochondrial number per cell (J). Scale bars: 2 μm (K) Representative flow cytometry plots showing expression of metabolic enzymes (ATP5A, IDH2, GLUT1, HK1, PKM, and LDH) in neutrophils. (L–M) Quantification of enzyme-positive neutrophils by proportion (L) and absolute number (M) across groups. Sample sizes: scRNA-seq, one sample per group; targeted metabolomics, n = 3 mice per group; Seahorse assays, n = 4 mice per group; TEM and flow cytometry, n = 5 mice per group. Data are presented as mean ± SEM. Statistical significance was determined by one-way ANOVA with post hoc testing. ∗P < 0.05, ∗∗P < 0.01, ∗∗∗P < 0.001, ∗∗∗∗P < 0.0001; ns, not significant.

Given the impact of Trem1 deficiency on myeloid cell recruitment, and the critical role of metabolic programs in regulating immune cell function during ischemia-reperfusion injury [33,34], we next investigated how Trem1 influences energy metabolism in neutrophils and macrophages. Single-cell RNA-seq–based differential pathway analysis of neutrophil clusters, comparing *Trem1−/−* and wild-type grafts, revealed significant enrichment of oxidative phosphorylation (OXPHOS), with glycolysis and the TCA cycle also affected (Fig. 4A–C). These findings suggested that Trem1 deficiency alters mitochondrial metabolism during neutrophil activation.

Targeted metabolomics of magnetically enriched neutrophils with >90 % purity, obtained using the same isolation strategy for both blood and graft-infiltrating cells and validated by flow cytometry (Fig. S8), demonstrated that under steady-state conditions in peripheral blood, Trem1 deficiency had no effect on glycolytic or TCA cycle intermediates(Fig. 4D). By contrast, in graft-infiltrating neutrophils after reperfusion, Trem1 deficiency led to accumulation of glycolytic and gluconeogenic intermediates (glucose-6-phosphate, fructose-6-phosphate, fructose-1,6-bisphosphate, sedoheptulose-7-phosphate, inosine) and reduction of TCA metabolites (α-ketoglutarate, fumarate) (Fig. 4D; Fig. S10). Notably, inosine levels have been previously reported to inversely correlate with lung injury after transplantation [20,35], consistent with the protective phenotype observed in *Trem1−/−* mice(Fig. 2).Functional assays corroborated these findings. Seahorse analyses showed no metabolic differences between circulating resting neutrophils from *Lysm*^*Cre*^*Trem1*^*fl*^ and *Trem1*^*fl*^ mice. However, in activated graft-infiltrating neutrophils, Trem1 deficiency decreased basal and maximal oxygen consumption and ATP production, while enhancing glycolytic capacity and reserve (Fig. 4E–H). Electron microscopy further revealed reduced mitochondrial numbers in activated *Lysm*^*Cre*^*Trem1*^*fl*^ neutrophils but not in circulating neutrophils (Fig. 4I and J). Flow cytometric metabolic profiling demonstrated downregulation of OXPHOS-related enzymes (ATP5A), TCA cycle enzymes (IDH2), and upregulation of glycolytic enzymes (GLUT1, HK1, PKM, LDH) in graft neutrophils from *Lysm*^*Cre*^*Trem1*^*fl*^ mice, whereas no differences were observed in blood neutrophils (Fig. 4K–M).

Bulk RNA-seq of sorted neutrophils confirmed these findings: no metabolic differences were detected in circulating neutrophils, but graft-infiltrating Trem1-deficient neutrophils showed marked enrichment of OXPHOS-related genes among differentially expressed pathways, in line with scRNA-seq results (Fig. S11). Together, these analyses indicate that Trem1 deficiency shifts neutrophil metabolism from oxidative phosphorylation toward glycolysis during lung reperfusion.

Macrophages exhibited distinct metabolic changes. Like neutrophils, Trem1 deficiency had no effect on steady-state macrophages in blood. However, in graft-infiltrating macrophages, Trem1 deficiency led to overall suppression of both glycolytic and oxidative metabolism, with consistent downregulation of ATP5A, IDH2, GLUT1, HK1, PKM, and LDH (Fig. S12).

These findings demonstrate that neutrophil activation during reperfusion requires enhanced metabolic activity, consistent with recent reports showing that activated neutrophils upregulate both glycolysis and oxidative phosphorylation to support effector functions [36]. Collectively, Trem1 deficiency induces distinct metabolic reprogramming in graft-infiltrating myeloid cells: neutrophils shift from oxidative phosphorylation toward glycolysis, whereas macrophages exhibit global suppression of energy metabolism.

### 3.6 Trem1 deficiency enhances AP-1 transcription factor activity in neutrophils and negatively regulates OXPHOS gene expression

**Fig. 5:**
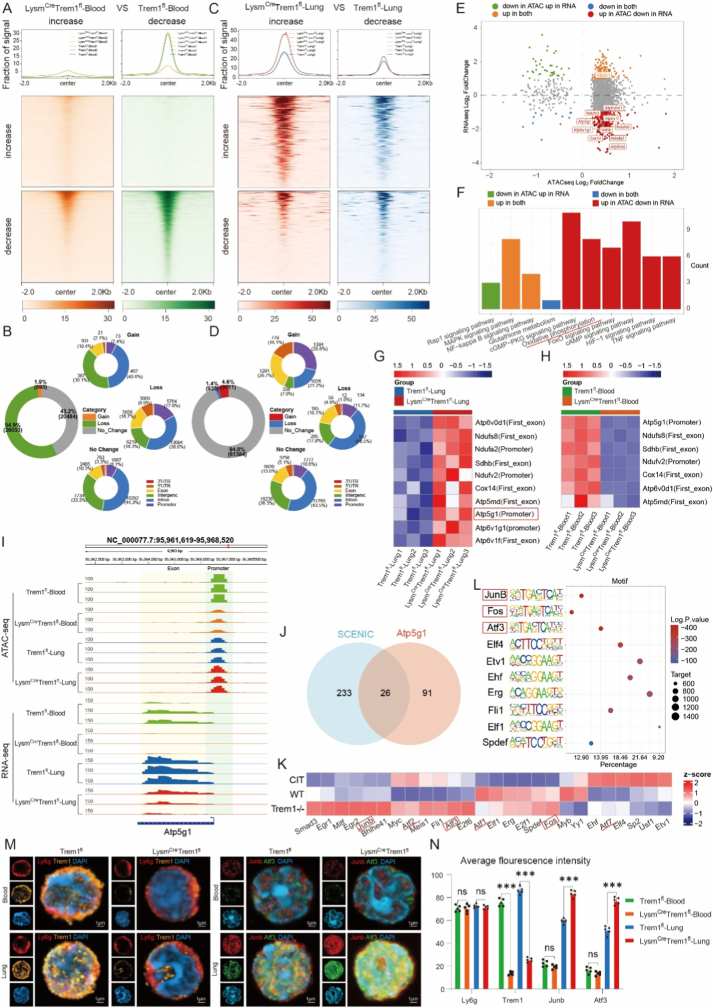
**Trem1 deletion enhances AP-1 family transcription factor expression in neutrophils.** (A–B) Chromatin accessibility in blood neutrophils (A) and graft neutrophils (B) from *Trem1*^*fl*^ versus *Lysm*^*Cre*^*Trem1*^*fl*^ mice, shown as aggregate signal plots (top) and heatmaps (bottom). (C–D) Pie charts summarizing gained and lost accessible regions in blood (C) and graft (D) neutrophils. (E–F) Four-quadrant volcano plot integrating ATAC-seq and bulk RNA-seq data from graft neutrophils (E), with pathway enrichment analysis of overlapping genes (F). (G–H) Heatmaps of oxidative phosphorylation–related genes showing differential chromatin accessibility in graft (G) and blood (H) neutrophils. (I) Representative IGV tracks displaying promoter and exon accessibility and RNA expression for Atp5g1. (J–L) Integrated analysis of transcription factor regulation: Venn diagram of SCENIC-inferred TFs and Atp5g1 motif-enriched TFs (J), heatmap of 26 shared TFs across groups (K), and motif enrichment of the top 10 TFs ranked by P value (L). (M) Confocal microscopy images showing Ly6g, Trem1, JunB, and Atf3 expression in blood and graft neutrophils from *Trem1*^*fl*^ and *Lysm*^*Cre*^*Trem1*^*fl*^ mice. Scale bars: 5 μm (overview), 1 μm (insets). (N) Quantification of average fluorescence intensity for Ly6g, Trem1, JunB, and Atf3 across groups. Sample sizes: ATAC-seq, n = 3 mice per group; SCENIC analysis, one sample per group; confocal microscopy and bulk RNA validation, n = 5 mice per group. Data are presented as mean ± SEM. Statistical significance determined by one-way ANOVA. ∗P < 0.05, ∗∗P < 0.01, ∗∗∗P < 0.001, ∗∗∗∗P < 0.0001; ns, not significant.

To further investigate the transcriptional regulation underlying OXPHOS alterations, we performed integrated ATAC-seq and bulk RNA-seq analyses on sorted neutrophils from blood (resting state) and graft tissue (activated state) (Fig. S8B). ATAC-seq revealed that Trem1 deficiency reduced chromatin accessibility in resting blood neutrophils, with loss of promoter and exon accessibility outweighing gains. By contrast, in graft-infiltrating neutrophils, Trem1 deficiency modestly increased chromatin accessibility, with more newly opened than closed promoter and exon regions (Fig. 5A–D). These findings indicate state-dependent regulation, whereby Trem1 deficiency decreases chromatin accessibility in resting neutrophils but increases it in activated graft neutrophils.

Comparison of wild-type neutrophils pre- and post-transplant demonstrated that migration into the graft was associated with overall reduced chromatin accessibility, despite upregulated OXPHOS gene expression at the RNA level (Figs. S11F, S13A, B). This suggests that transcriptional regulation of OXPHOS genes during neutrophil activation may involve compensatory or inhibitory mechanisms.

Indeed, joint ATAC-seq and RNA-seq analyses of graft neutrophils revealed that OXPHOS-related genes in Trem1-deficient neutrophils showed increased promoter accessibility but decreased RNA expression (Fig. 5E–H). For example, Atp5g1 exhibited reduced promoter accessibility and RNA expression in resting blood neutrophils, but paradoxically increased promoter accessibility with reduced transcription in graft neutrophils lacking Trem1 (Fig. 5I).

Motif analysis of OXPHOS gene promoters, combined with SCENIC analysis of scRNA-seq data, identified 26 candidate transcription factors, among which AP-1 family members (JunB, Fos, Atf3, Atf2, Atf1, Atf7) were most significantly enriched (Fig. 5J–L), consistent with prior reports that AP-1 complexes orchestrate immune cell activation and metabolic adaptation [37,38].Immunofluorescence staining further confirmed increased expression of JunB and Atf3 in graft neutrophils from *Lysm*^*Cre*^*Trem1*^*fl*^ mice, whereas no differences were observed in circulating blood neutrophils (Fig. 5M and N). These findings align with recent multi-omics studies and functional reports showing that AP-1 transcription factors, including c-Fos, promote neutrophil activation, metabolic reprogramming, and NET formation [39].

Together, these results suggest that Trem1 deficiency differentially regulates neutrophil chromatin accessibility depending on activation state, and that AP-1 family transcription factors are upregulated in graft-infiltrating neutrophils, where they may act as negative regulators of OXPHOS gene transcription.

### 3.7 Pharmacologic inhibition of Atf3 restores OXPHOS activity, neutrophil recruitment, and graft injury despite Trem1 deficiency

Previous studies have shown that AP-1 transcription factors are highly expressed in neutrophils under inflammatory conditions, and that Atf3 deficiency exacerbates ischemia-reperfusion–induced organ injury while potentially acting as a negative regulator of oxidative phosphorylation [[40], [41], [42], [43]]. Based on our findings that Trem1 deficiency suppressed OXPHOS while upregulating Atf3 in activated graft neutrophils, we hypothesized that Trem1 limits neutrophil recruitment by promoting Atf3-mediated repression of OXPHOS.

**Fig. 6:**
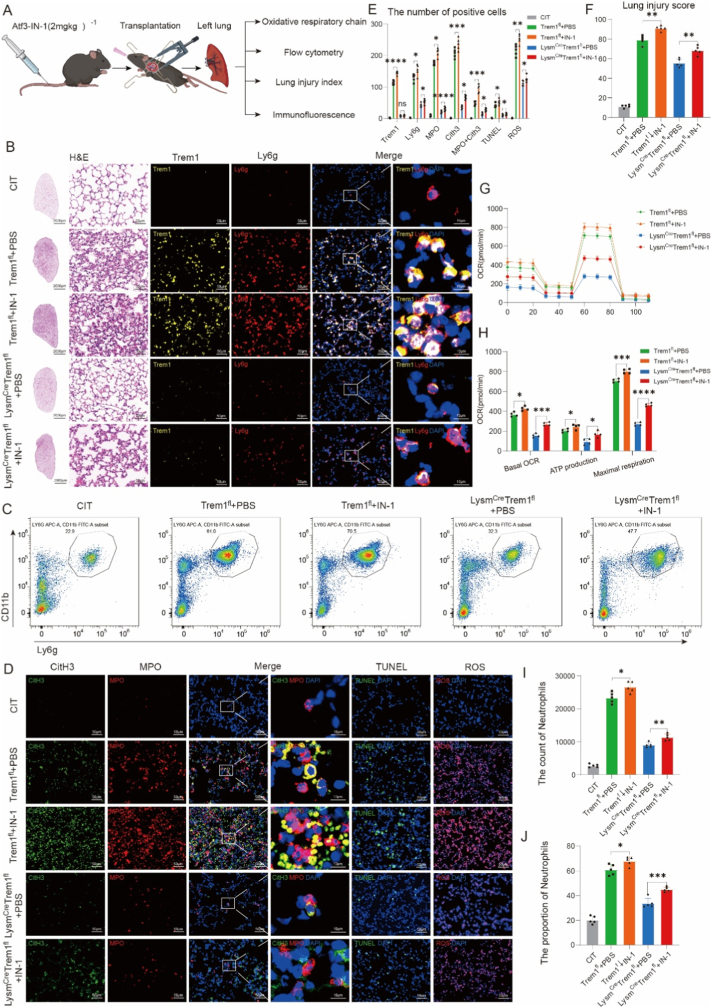
**Pharmacologic activation of OXPHOS restores neutrophil recruitment despite Trem1 deficiency.** (A) Schematic of experimental design. *Trem1*^*fl*^ and *Lysm*^*Cre*^*Trem1*^*fl*^ mice were pretreated with the Atf3 inhibitor IN-1 (10 mg/kg, i.p.) prior to lung transplantation and analyzed after 4 h of reperfusion. Endpoints included histology, immunofluorescence, flow cytometry, Seahorse metabolic assays, and lung injury scoring. (B) Representative H&E staining and immunofluorescence for Trem1 and Ly6g in grafts from indicated groups. Scale bars: 2000 μm (overview), 50 μm (high magnification). (C) Flow cytometry plots showing frequencies of CD11b^+^Ly6g^+^ neutrophils. (D) Immunofluorescence of CitH3, MPO, TUNEL, and ROS in graft sections. Scale bars: 50 μm (E) Quantification of Trem1^+^, Ly6G^+^, CitH3^+^, MPO^+^, TUNEL^+^, and ROS^+^ cells, including quantification of MPO^+^CitH3^+^ double-positive neutrophils as an index of NET formation. (F) Lung injury scores derived from H&E-stained grafts. (G–H) Seahorse assays of neutrophils isolated from grafts showing OCR traces (G) and quantification of basal OCR, ATP production, and maximal respiration (H). (I–J) Flow cytometry quantification of neutrophil counts (I) and percentages (J) in grafts. Sample sizes: Seahorse assays, n = 4 mice per group; flow cytometry, immunofluorescence, and histology, n = 5 mice per group. Data are expressed as mean ± SEM. Statistical significance determined by one-way ANOVA. ∗P < 0.05, ∗∗P < 0.01, ∗∗∗P < 0.001, ∗∗∗∗P < 0.0001; ns, not significant.

To test this hypothesis, we utilized IN-1, a pharmacologic inhibitor previously shown to suppress Atf3 expression in neuronal ischemia-reperfusion models [44]. IN-1 was administered by tail vein injection prior to transplantation (Fig. 6A). Treatment with IN-1 significantly reduced Atf3 expression in graft-infiltrating neutrophils (Fig. S14A and B). Histology, immunofluorescence, and flow cytometry demonstrated that IN-1 treatment increased neutrophil recruitment to the graft, aggravated lung injury, and enhanced NET formation in both *Trem1*^*fl*^ and *Lysm*^*Cre*^*Trem1*^*fl*^ recipients (Fig. 6B–F, I, J). Metabolic profiling further revealed that IN-1 restored oxidative phosphorylation activity in graft neutrophils, as reflected by increased oxygen consumption and ATP production (Fig. 6G and H).

Together, these results demonstrate that Atf3 negatively regulates OXPHOS in activated neutrophils, and that pharmacologic inhibition of Atf3 abrogates the protective effects of Trem1 deficiency by restoring neutrophil metabolic activity, recruitment, and tissue injury after lung transplantation.

### 3.8 TREM1+ neutrophil recruitment correlates with PGD severity in human lung transplantation

Analysis of human lung tissue revealed increased TREM1 expression after reperfusion, with TREM1 predominantly localized to neutrophils (Fig. S1B–D). We therefore examined whether recruitment of TREM1^+^ neutrophils was associated with the severity of primary graft dysfunction (PGD) [[45], [46], [47]].

**Fig. 7:**
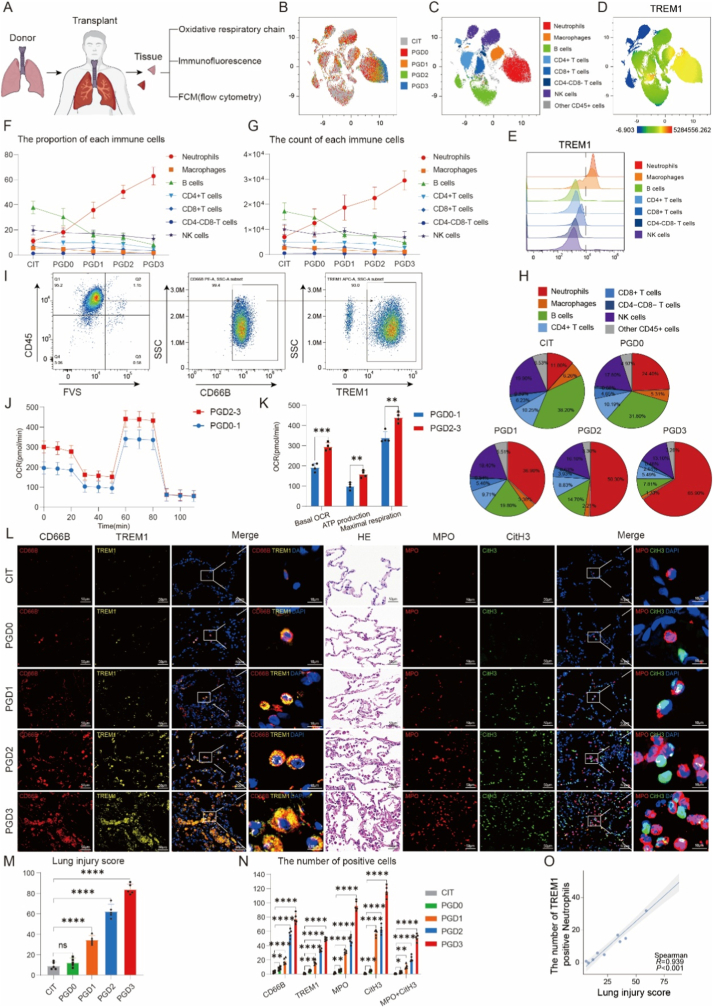
**Accumulation of TREM1^+^ neutrophils correlates with PGD severity in human lung transplantation.** (A) Schematic of study design showing collection of human lung graft tissue across PGD grades (n = 25 recipients; 5 per group for PGD0–3 and CIT). (B–C) UMAP plots showing clustering of immune cell populations from lung grafts across PGD grades. (D–E) TREM1 expression across immune subsets displayed as UMAP projection (D) and density plots (E). (F–G) Quantification of immune cell proportions (F) and absolute counts (G) across PGD grades. (H) Pie charts showing relative distribution of CD45^+^ immune subsets in CIT and PGD groups. (I) Representative flow cytometry plots confirming purity of magnetically sorted CD66B^+^ neutrophils from human grafts. (J–K) Seahorse analysis of neutrophils isolated from PGD0–1 versus PGD2–3 grafts, showing OCR traces (J) and quantification of basal OCR, ATP production, and maximal respiration (K). (L) Representative immunofluorescence staining of graft sections for CD66B, TREM1, MPO, and CitH3, together with H&E staining. Scale bars: 50 μm (IF), 2000 μm (H&E overview). (M) Lung injury scores across PGD grades. (N) Quantification of CD66B^+^, TREM1^+^, MPO^+^, and CitH3^+^ cells in human lung graft sections across PGD grades. For NET assessment, MPO and CitH3 were used as complementary markers; MPO^+^CitH3^+^ double-positive cells were defined as NET-forming cells, and MPO-only and CitH3-only single-positive cells were quantified separately. Positive cells were counted using ImageJ from five randomly selected high-power fields per section. (O) Correlation between numbers of TREM1^+^ neutrophils and lung injury scores (Spearman's R = 0.930, P < 0.001). Sample sizes: Seahorse assays, n = 4 patients per group (PGD0–1 vs PGD2–3); immunofluorescence, flow cytometry, and histology, n = 5 per group. Data are expressed as mean ± SEM. Statistical significance was assessed using one-way ANOVA or Spearman correlation. ∗P < 0.05, ∗∗P < 0.01, ∗∗∗P < 0.001, ∗∗∗∗P < 0.0001; ns, not significant.

Lung tissues from 25 recipients were analyzed, including donor lungs prior to transplantation (CIT, *n* = 5) and reperfused grafts stratified by PGD grade (PGD0–3, *n* = 5 per group) (Fig. 7A). Flow cytometric analysis of single-cell suspensions demonstrated that TREM1 expression was largely restricted to myeloid cells, with neutrophils representing the dominant recruited immune population (Fig. 7B–H).Consistent with murine findings, compositional analysis further revealed that neutrophils constituted the predominant cell subset within the TREM1^+^ immune cell compartment across all PGD grades (Fig. S15).

Notably, both total neutrophil infiltration and accumulation of TREM1^+^ neutrophils increased progressively with PGD severity (Fig. 7B–H). Purified neutrophils isolated from PGD2–3 grafts exhibited significantly higher oxidative respiratory capacity compared with those from PGD0–1 grafts, as assessed by Seahorse analysis (Fig. 7J and K). Histological examination and immunofluorescence further confirmed exacerbated tissue injury, increased neutrophil accumulation, and enhanced neutrophil extracellular trap (NET) formation in higher-grade PGD grafts (Fig. 7L–O).

Collectively, these findings demonstrate that **enrichment of TREM1^+^ neutrophils**, rather than global TREM1 expression across immune cell types, is closely associated with PGD severity, thereby linking experimental mechanisms identified in murine models to clinically relevant outcomes in human lung transplantation.

## 4 Discussion

Ischemia-reperfusion injury is a central driver of primary graft dysfunction (PGD) after lung transplantation [48,49], yet the molecular regulators that link inflammatory signaling to immune cell metabolism remain poorly defined. In this study, we identify Trem1 as a critical node at the intersection of inflammation and metabolic regulation in myeloid cells. Using a clinically relevant murine lung transplantation model, we show that Trem1 deficiency markedly attenuates early graft injury by reducing neutrophil recruitment, dampening NET formation, and mitigating tissue damage. Mechanistically, Trem1 deletion suppressed oxidative phosphorylation (OXPHOS) in graft-infiltrating neutrophils through upregulation of the transcriptional repressor Atf3, an AP-1 family member, thereby limiting the energy supply required for neutrophil accumulation in injured lungs. Importantly, analysis of human transplant samples demonstrated that the abundance of TREM1+ neutrophils correlated directly with PGD severity and was accompanied by enhanced OXPHOS activity, establishing clinical relevance for this regulatory axis.

These findings advance our understanding of Trem1 beyond its classical role as an amplifier of inflammatory responses [50]. Whereas previous work has focused primarily on Trem1-mediated cytokine release and leukocyte activation [3], our results reveal a previously unrecognized function of Trem1 in metabolic control of neutrophils. By delineating a Trem1–Atf3–OXPHOS axis that governs neutrophil recruitment and injury severity, our study uncovers a mechanistic link between inflammatory signaling and immunometabolic adaptation during lung transplantation.

Beyond modulating leukocyte recruitment, Trem1 also shapes the functional polarization of myeloid cells [[51], [52], [53], [54]]. In our prior work using an orthotopic tracheal transplantation model, we demonstrated that Trem1 deficiency mitigated obliterative bronchiolitis by suppressing Nod-like receptor signaling and restraining macrophage proinflammatory differentiation [55]. Extending these findings to the lung transplantation setting, we now show that Trem1 ablation alters the differentiation trajectories of both neutrophils and macrophages. Single-cell pseudotime analysis revealed that Trem1-deficient neutrophils in graft tissue exhibited a dual polarization pattern, with subsets skewing toward both proinflammatory and anti-inflammatory fates, consistent with upregulation of Ccl3, IL1β, NF-κB, Nlrp3, and Tnf on one hand, and compensatory anti-inflammatory signatures on the other [56] (Fig. S16–I). Flow cytometry validation clarified this paradox: although Trem1 deletion promoted divergent polarization programs, the overall number of infiltrating neutrophils was markedly reduced, leading to a net attenuation of inflammatory burden in the graft (Fig. S16J–O).

Macrophages displayed a distinct pattern. In lung allografts, Trem1 deletion constrained M1 polarization while promoting M2 differentiation (Fig. S17A–D), in agreement with our previous findings in tracheal transplantation–induced obliterative bronchiolitis [55]. Together, these observations establish that Trem1 governs both the quantity and the quality of myeloid cell responses in transplantation: it amplifies infiltration while simultaneously directing cells toward proinflammatory functional states. The convergence of these dual regulatory axes likely explains the potent effect of Trem1 deletion on mitigating ischemia-reperfusion injury and PGD development.

Our data further underscore a fundamental divergence in metabolic programming between neutrophils and macrophages during lung ischemia-reperfusion. Neutrophils recruited into graft tissue displayed markedly increased metabolic activity compared with circulating counterparts, as evidenced by elevated glycolytic intermediates, enhanced glycolytic capacity, and increased mitochondrial activity (Fig. 4D–M). This metabolic shift coincided with pseudotime analyses showing an enrichment of proinflammatory transcriptional signatures, suggesting that heightened glycolytic flux may bias neutrophils toward proinflammatory polarization within injured lungs.

By contrast, macrophages demonstrated an opposite trend. Trem1 deficiency reduced overall macrophage infiltration, and metabolic profiling revealed that graft-infiltrating macrophages exhibited attenuated expression of key metabolic enzymes, including GLUT1, ATP5A, IDH2, and glycolytic effectors, compared with blood-derived macrophages (Fig. S9E–G). Given the established role of glycolysis in sustaining proinflammatory M1 polarization, these findings imply that reduced glucose uptake and impaired glycolytic capacity may limit proinflammatory differentiation while constraining overall metabolic activity in macrophages [9,57]. The greater downregulation of GLUT1 in macrophages relative to neutrophils suggests that glucose transporter expression may represent a decisive checkpoint controlling the divergent metabolic adaptations of myeloid subsets during reperfusion injury [58,59].

Our integrated ATAC-seq and transcriptomic analyses further uncovered an unexpected layer of regulation, linking Trem1 signaling to chromatin dynamics in myeloid cells. In resting neutrophils from the circulation, Trem1 deletion reduced global chromatin accessibility, with preferential loss of promoter and exonic regions, consistent with a more quiescent state (Fig. 5A–D). By contrast, in graft-infiltrating neutrophils, Trem1 deficiency led to modestly increased chromatin accessibility, particularly at promoter and exon regions, despite a concurrent downregulation of oxidative phosphorylation (OXPHOS)-related transcripts (Fig. 5E–H). This apparent discordance—enhanced chromatin openness yet reduced transcription of OXPHOS genes—suggests the existence of negative regulatory mechanisms at the transcriptional level, reminiscent of recent observations that metabolic intermediates can shape chromatin architecture and, in some cases, decouple accessibility from gene expression outputs [60,61].

Moreover, we observed that the impact of Trem1 on myeloid metabolism was state-dependent: in circulating, resting neutrophils, Trem1 loss had minimal effects, whereas in activated graft-infiltrating neutrophils, Trem1 deficiency resulted in reduced OXPHOS activity. Concomitantly, transcription factor analyses revealed selective induction of AP-1 family members—including JUNB, FOS, ATF3, and ATF2—in Trem1-deficient neutrophils within transplanted lungs (Fig. 5I–L). Prior reports have established that AP-1 complexes orchestrate immune cell activation and metabolic reprogramming [62], while ATF3 has been implicated in modulating inflammatory injury responses [43,63]. Our data suggest that Trem1 regulates neutrophil OXPHOS through an Atf3-dependent inhibitory pathway, thereby limiting mitochondrial metabolism and restraining neutrophil accumulation during reperfusion.

Our analysis of human lung transplant specimens further substantiates the translational significance of the Trem1–Atf3–OXPHOS axis. We observed that TREM1 expression was enriched in myeloid populations, particularly neutrophils, within transplanted lungs, and that the frequency of TREM1^+^ neutrophils increased in parallel with PGD severity (Fig. 7B–H). Moreover, metabolic profiling of graft-infiltrating neutrophils revealed that higher PGD grades were associated with augmented OXPHOS activity (Fig. 7J and K), consistent with the concept that heightened mitochondrial metabolism supports sustained neutrophil activation and tissue injury. These clinical findings mirror our experimental data and point to the Trem1–Atf3–OXPHOS pathway as both a potential biomarker of PGD progression and a promising therapeutic target to mitigate ischemia-reperfusion–driven lung injury.

Our study has several limitations. First, the number of clinical lung transplant specimens was relatively modest, which may constrain the generalizability of our findings. Second, although we employed the pharmacologic inhibitor IN-1 to interrogate ATF3 function, its specificity has not been fully established, and off-target effects cannot be excluded. Third, while our multi-omics analyses highlighted a central role for the Trem1–Atf3–OXPHOS axis, it remains possible that additional transcriptional regulators or metabolic pathways contribute to the observed effects on myeloid cell recruitment and polarization. Finally, our human validation focused on early PGD; whether Trem1-dependent metabolic and immunologic alterations persist and influence long-term graft outcomes warrants further investigation in larger, longitudinal cohorts.

In conclusion, our study identifies Trem1 as a key nexus linking inflammatory signaling with immunometabolic reprogramming in lung ischemia–reperfusion injury and PGD. We show that Trem1 deficiency mitigates graft damage by limiting neutrophil infiltration and NET formation, an effect associated with *Atf3*-dependent repression of OXPHOS. Notably, Trem1 differentially shapes neutrophil and macrophage polarization, thereby influencing both the magnitude and quality of myeloid responses. Validation in human lung transplant samples, where TREM1^+^ neutrophil accumulation and elevated OXPHOS correlate with PGD severity, underscores the translational significance of these findings. Together, our data reveal a previously unrecognized Trem1–Atf3–OXPHOS axis and highlight its potential as both a biomarker and a therapeutic target in PGD.

## 5 Conclusion

TREM1 drives neutrophil recruitment and oxidative metabolism during lung ischemia-reperfusion injury, and its inhibition mitigates inflammation and primary graft dysfunction, highlighting TREM1 as a potential therapeutic target in lung transplantation.

## Funding

This work was supported by the National Science and Technology Major Project of China (grant number 2024ZD0529005, to Sihua Wang) and the 10.13039/501100001809National Natural Science Foundation of China (grant numbers 82570141 to Sihua Wang and 82470107 to Guangjian Zhang).

## CRediT authorship contribution statement

**Fengjing Yang:** Conceptualization, Data curation, Formal analysis, Investigation, Methodology, Project administration, Resources, Software, Supervision, Validation, Visualization, Writing – original draft, Writing – review & editing. **Song Tong:** Data curation, Formal analysis, Visualization. **Junhao Wan:** Investigation, Writing – original draft, Writing – review & editing. **Yixing Li:** Data curation, Investigation. **Jiani Gao:** Resources, Software. **Yan Sun:** Supervision, Validation. **Xiangfu Sun:** Formal analysis, Software. **Huikang Fu:** Software, Validation, Visualization. **Wenzhuo Luo:** Software, Validation. **Jiayang Xu:** Formal analysis, Software. **Ting Zhou:** Funding acquisition, Supervision. **Sowe Babou:** Formal analysis, Resources. **Junqi Wu:** Investigation, Supervision. **Guangjian Zhang:** Conceptualization, Data curation, Funding acquisition. **Chang Chen:** Methodology, Visualization. **Sihua Wang:** Funding acquisition, Writing – original draft, Writing – review & editing.

## Declaration of competing interest

The authors declare no competing interests.
