# Supplementary material for: Trem1 regulates neutrophil metabolism and recruitment in lung ischemia-reperfusion injury
Source: Redox Biol. 2026 Jan 14;92:104026. doi: 10.1016/j.redox.2026.104026 (PMC13019080; doi:10.1016/j.redox.2026.104026)
Supplement: Multimedia component 1 [file mmc1.docx]

**Supplemental information**

**Trem1 regulates neutrophil metabolism and recruitment in lung ischemia-reperfusion injury**

**Fengjing Yang, Song Tong, Junhao Wan, Yixing Li, Jiani Gao,Yan Sun,Xiangfu Sun, Huikang Fu, Luowen Zhuo, Jiayang Xu, Ting Zhou, SOWE BABOU, Junqi Wu, Guangjian Zhang,** **Chang Chen, Sihua Wang**


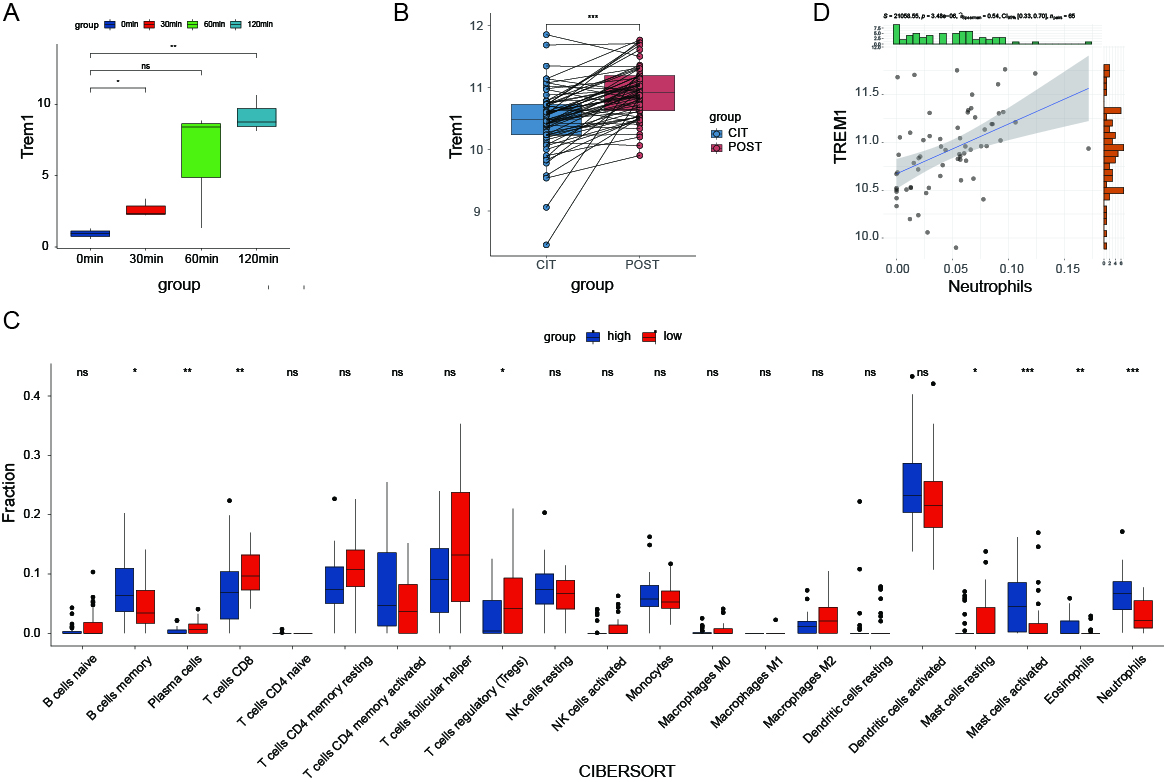


**Figure S1. Trem1 expression increases with reperfusion and correlates with neutrophil infiltration. Related to Figure 1.**

(A) Analysis of GSE203238 (mouse hilar clamp model) showing box plots of Trem1 expression at indicated reperfusion time points (0, 30, 60, and 120 min). n = 3 per group.
(B) Analysis of GSE145989 comparing paired human lung tissue samples before (CIT) and after transplantation (POST), showing significantly higher TREM1 expression post-transplant (n = 52 pairs).
(C) CIBERSORT deconvolution of GSE145989 stratified by high versus low TREM1 expression, revealing differential immune cell fractions.
(D) Correlation analysis of TREM1 expression with neutrophil fraction estimated by CIBERSORT in GSE145989 (Spearman’s R = 0.54, P < 0.001).

Data are shown as mean ± SEM or paired values, as appropriate. Statistical significance was determined by one-way ANOVA (A), paired Student’s t test (B), or Spearman correlation (D). *P < 0.05, **P < 0.01, ***P < 0.001, ns = not significant.


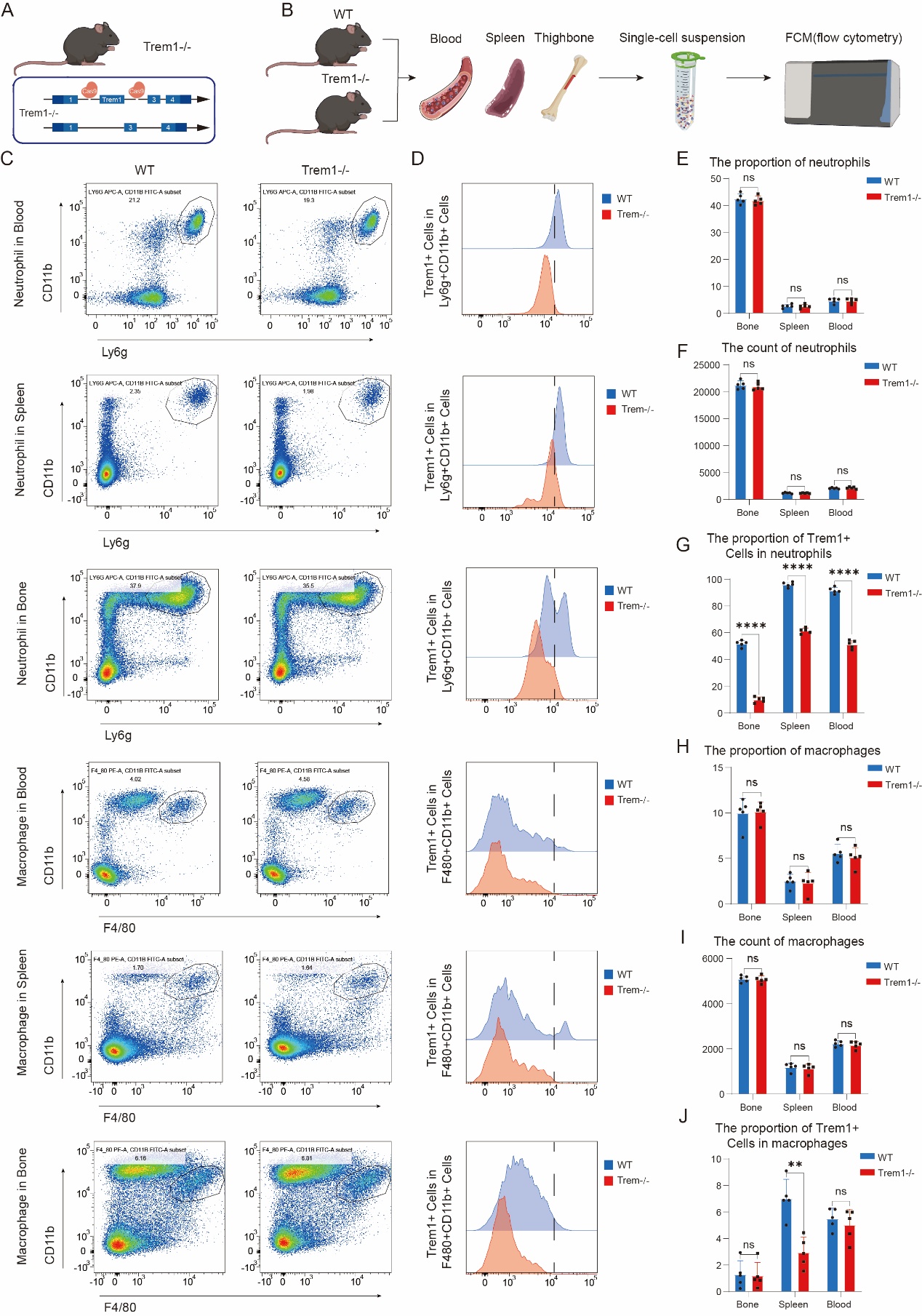


**Figure S2. Generation of *Trem1-/-* mice and validation of knockout efficiency.** **Related to Figure 2.**

(A) Schematic diagram of Trem1 gene targeting strategy used to generate *Trem1-/-* mice.
(B) Workflow of sample preparation and flow cytometry: spleen, bone marrow, and peripheral blood were collected from WT and *Trem1-/-* mice, processed into single-cell suspensions, and analyzed by flow cytometry.
(C) Representative flow cytometry plots showing neutrophils (Ly6G⁺CD11b⁺) and macrophages (F4/80⁺CD11b⁺) from blood, spleen, and bone marrow of WT and *Trem1-/-* mice.
(D) Histograms comparing Trem1 expression in neutrophils and macrophages across tissues.
(E, F) Quantification of neutrophil proportion (E) and absolute count (F) in blood, spleen, and bone marrow.
(G) Proportion of Trem1⁺ cells among neutrophils.
(H, I) Quantification of macrophage proportion (H) and absolute count (I) in blood, spleen, and bone marrow.
(J) Proportion of Trem1⁺ cells among macrophages.

Data are shown as mean ± SEM (n = 5 per group). Statistical analysis was performed using Student’s t test. **P < 0.01, ****P < 0.0001; ns = not significant.


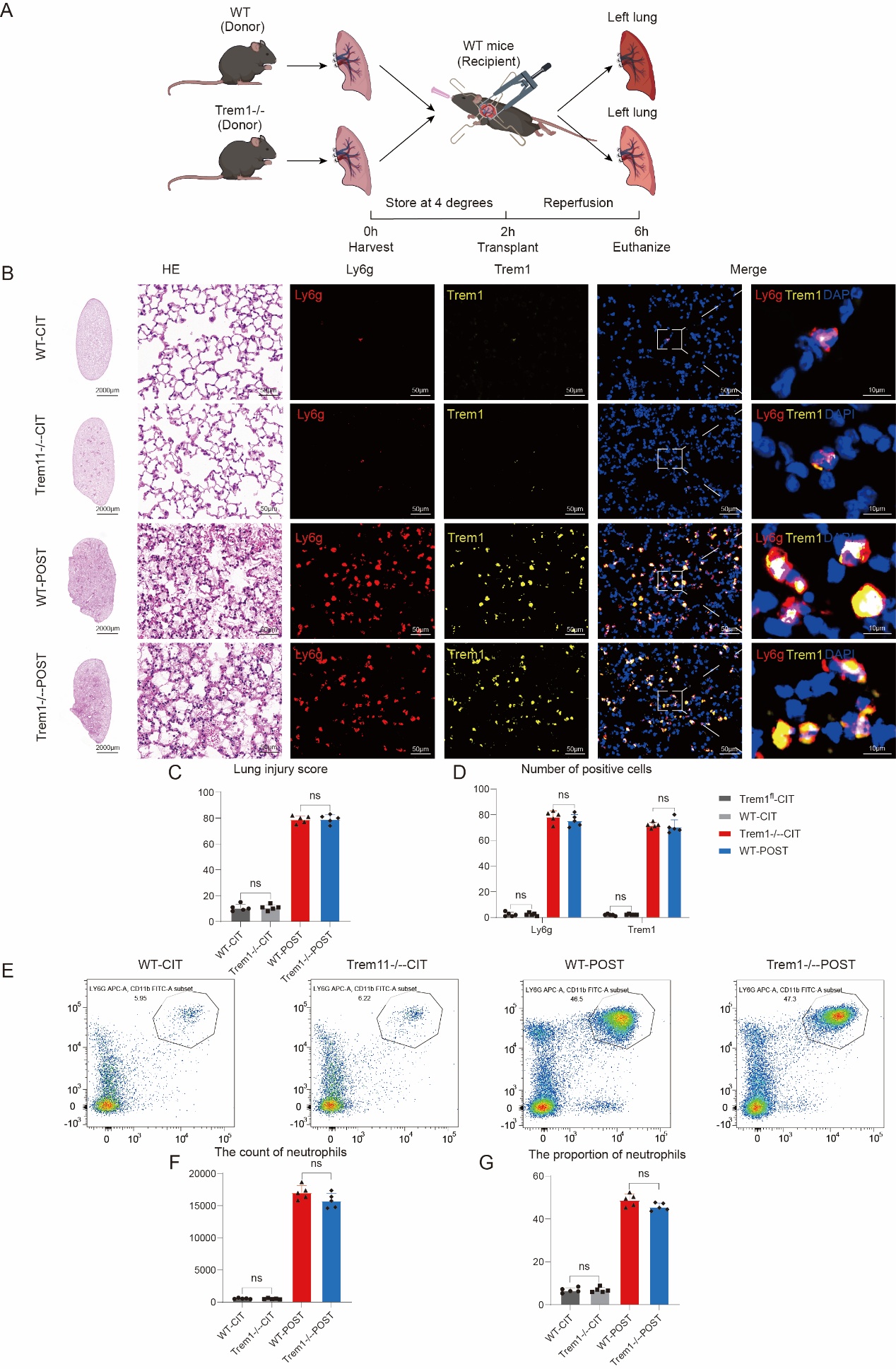


**Figure S3.Transplantation of *Trem1-/-* donor lungs into WT recipients does not markedly attenuate lung ischemia–reperfusion injury.Related to Figure 2.**

(A) Schematic illustration of the experimental design in which lungs from WT or *Trem1-/-* donor mice were transplanted into WT recipient mice, followed by cold ischemia and reperfusion.

(B) Representative hematoxylin and eosin (H&E) staining and immunofluorescence images of donor lungs (CIT) and transplanted lungs after reperfusion (POST), showing Ly6G (red) and Trem1 (yellow) expression, with nuclei counterstained with DAPI (blue).

(C) Quantification of lung injury scores based on H&E-stained sections.

(D) Quantification of Ly6G⁺ neutrophils and Trem1⁺ cells in lung tissue based on immunofluorescence analysis.

(E) Representative flow cytometry plots showing neutrophils (CD45⁺CD11b⁺Ly6G⁺) in donor lungs and transplanted lungs.

(F) Quantification of absolute neutrophil counts determined by flow cytometry.

(G) Quantification of the proportion of neutrophils among total lung immune cells determined by flow cytometry.

Data are presented as mean ± SEM. Statistical significance was assessed using one-way ANOVA; ns, not significant.


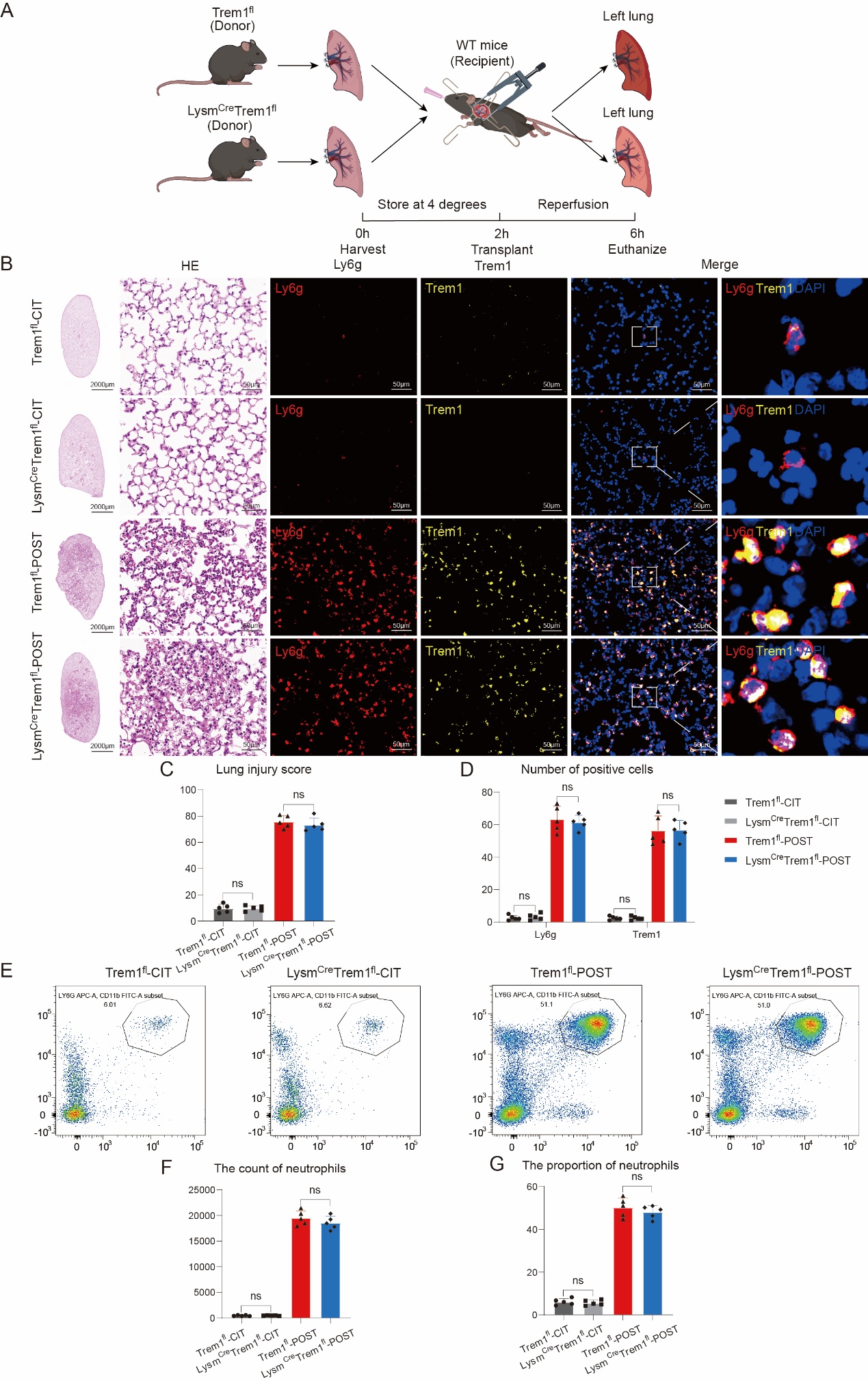


**Figure S4.** **Transplantation of lungs from *Lysm^Cre^Trem1^fl^* donors does not significantly attenuate lung ischemia–reperfusion injury in wild-type recipients. Related to Figure 2.**

(A) Schematic illustration of orthotopic lung transplantation, in which donor lungs from *Trem1^fl^* or *Lysm^Cre^Trem1^fl^* mice were transplanted into wild-type (WT) recipient mice, followed by cold ischemia and reperfusion.

(B) Representative hematoxylin and eosin (H&E) staining and immunofluorescence staining for Ly6G and Trem1 in donor lungs under cold ischemia (CIT) conditions and in graft lungs after reperfusion (POST). Nuclei were counterstained with DAPI.

(C) Quantification of lung injury scores based on H&E-stained sections in the indicated groups.

(D) Quantification of Ly6G⁺ neutrophils and Trem1⁺ cells in lung sections based on immunofluorescence analysis.

(E) Representative flow cytometry plots showing neutrophils (CD11b⁺Ly6G⁺) in donor lungs and transplanted lungs after reperfusion.

(F) Quantification of absolute neutrophil counts in lung tissues determined by flow cytometry.

(G) Quantification of neutrophil proportions among total CD45⁺ immune cells in lung tissues determined by flow cytometry.

Data are presented as mean ± SEM. Statistical significance was determined by one-way ANOVA. ns, not significant.


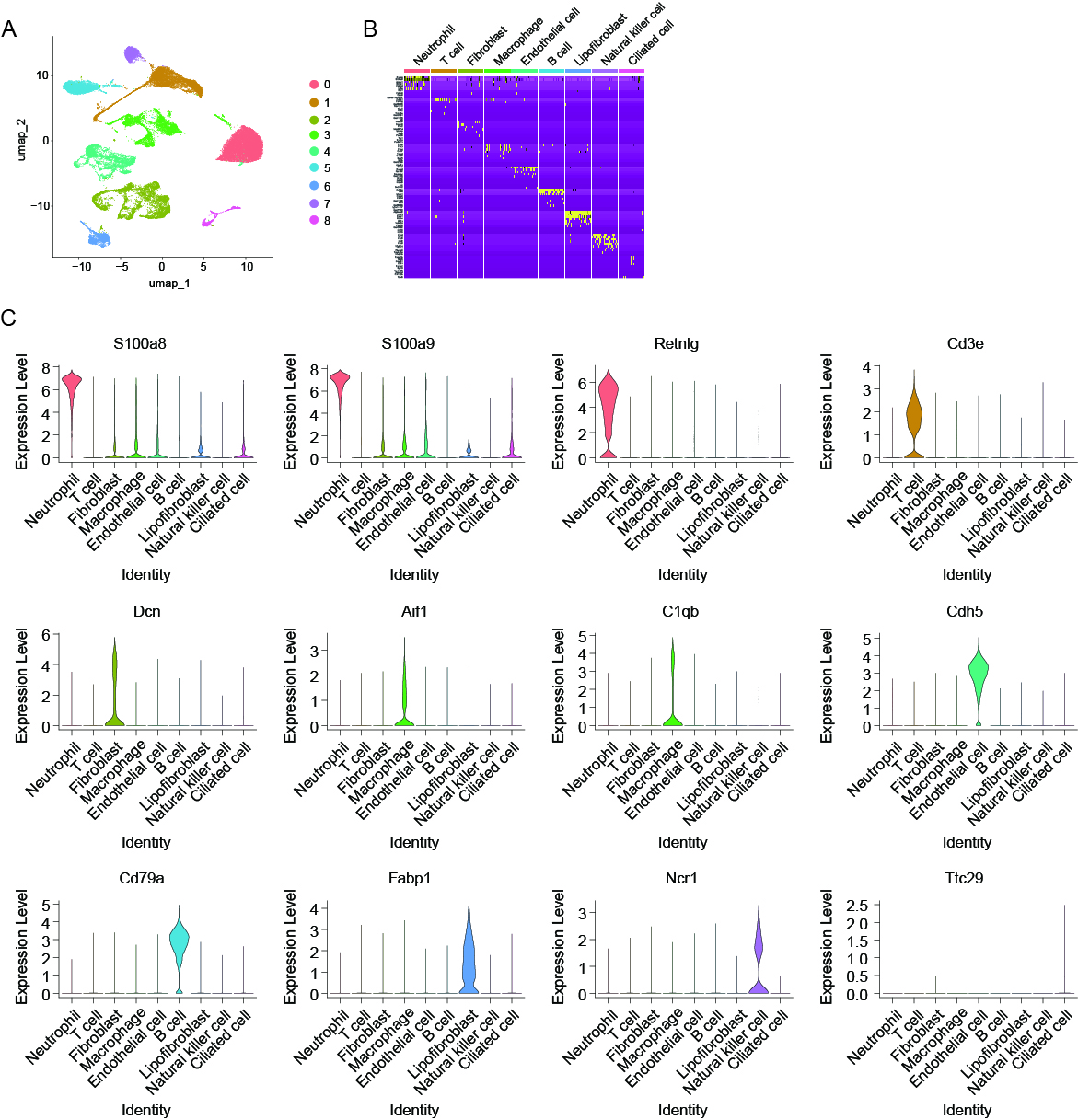


**Figure S5. Identification of major cell clusters and representative marker genes in scRNA-seq. Related to Figure 3.**

(A) UMAP projection of lung single-cell transcriptomes showing major immune and stromal populations.
(B) Heatmap of the top 10 differentially expressed genes (avg_log2FC) defining each cluster.
(C) Violin plots of representative marker genes for selected cell subsets, including neutrophils (S100a8, S100a9, Retnlg), T cells (Cd3e), fibroblasts (Dcn), macrophages (Aif1, C1qb), endothelial cells (Cdh5), B cells (Cd79a), epithelial cells (Fabp1), NK cells (Ncr1), and ciliated cells (Ttc29).


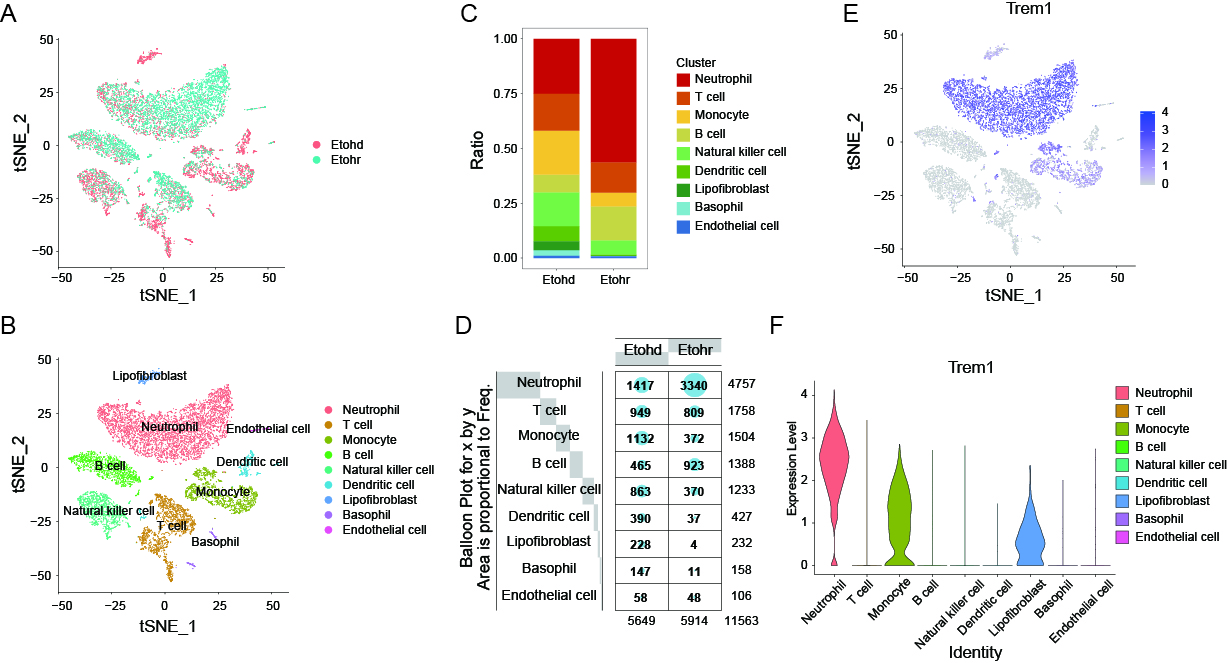


**Figure S6. Trem1 is predominantly expressed in myeloid cells within post-transplant lung tissue. Related to Figure 3.**

(A) tSNE projection of lung single-cell transcriptomes from the GSE237110 dataset, stratified by sample group (Et0hd vs Et0hr).
(B) Cell type annotation of major clusters, including neutrophils, T cells, monocytes, B cells, natural killer cells, dendritic cells, lipofibroblasts, basophils, and endothelial cells.
(C) Relative proportions of each immune and stromal cluster across groups, shown as stacked bar plots.
(D) Balloon plots and counts of individual cell types across groups.
(E) tSNE projection showing the distribution of Trem1 expression across lung cell populations.
(F) Violin plots depicting Trem1 expression across annotated cell subsets, with highest expression observed in neutrophils and monocytes.


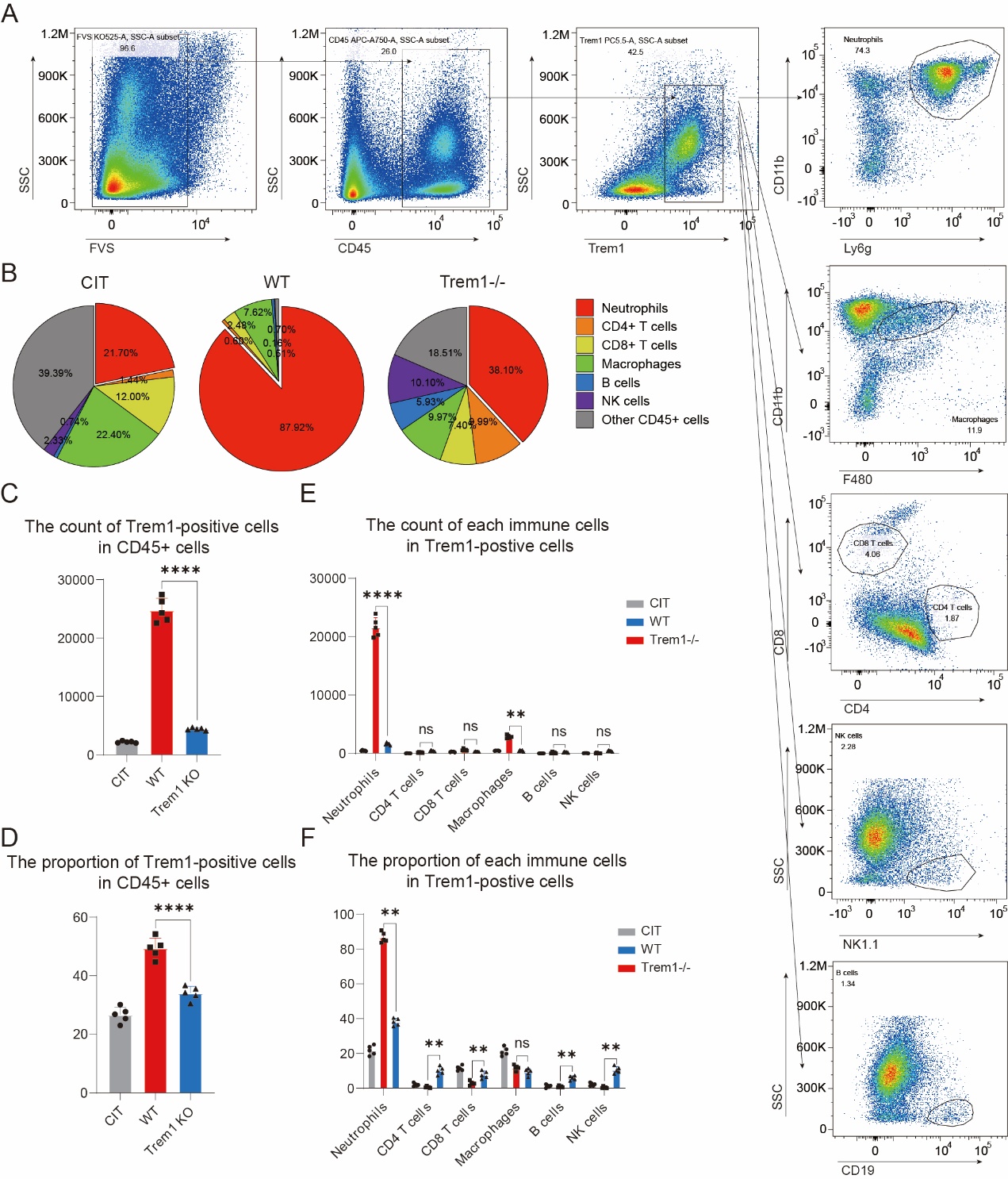


**Figure S7. Trem1-positive cells in lung tissue are predominantly of myeloid origin, with neutrophils representing the major cellular subset. Related to Figure 3.**

(A) Flow cytometry gating strategy for the identification of Trem1⁺ immune cell populations in lung tissue, including neutrophils (CD45⁺CD11b⁺Ly6G⁺), macrophages (CD45⁺CD11b⁺F4/80⁺), CD4⁺ T cells, CD8⁺ T cells, B cells, and NK cells.

(B) Pie charts showing the relative composition of immune cell subsets among Trem1⁺ cells in lung tissues under cold ischemia (CIT), wild-type (WT), and *Trem1-/-* conditions.

(C) Quantification of the absolute number of Trem1⁺ cells among total CD45⁺ immune cells in lung tissues from the indicated groups.

(D) Quantification of the proportion of Trem1⁺ cells among total CD45⁺ immune cells in lung tissues from the indicated groups.

(E) Quantification of the proportion of each immune cell subset within the Trem1⁺ cell population.

(F) Quantification of the absolute number of each immune cell subset within the Trem1⁺ cell population.

Data are presented as mean ± SEM. Statistical significance was determined by one-way ANOVA. **P < 0.01, ****P < 0.0001; ns, not significant.


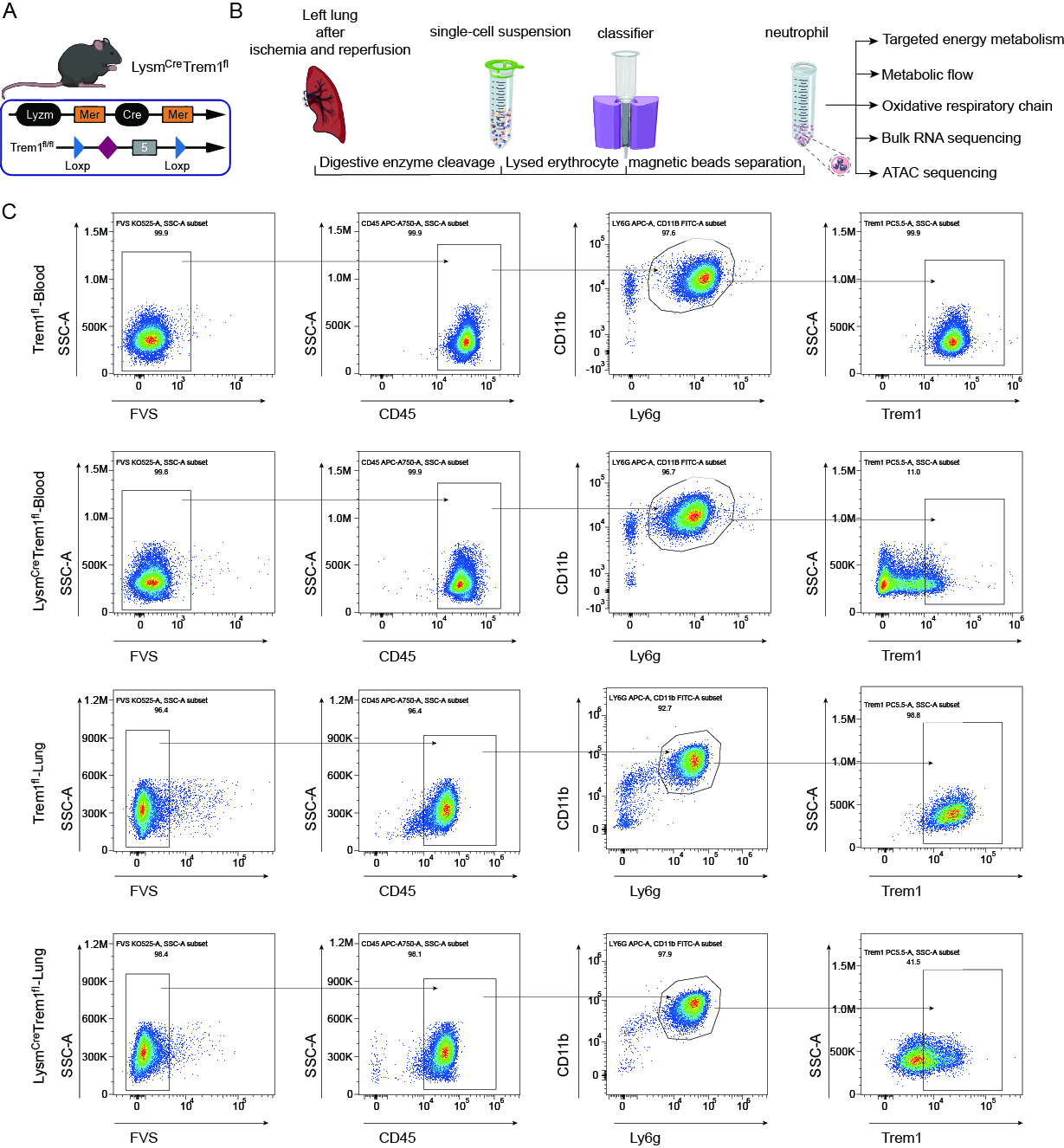


**Figure S8. Generation of myeloid-specific Trem1 conditional knockout mice and validation of neutrophil isolation. Related to Figure 4.**

(A) Schematic illustration of the strategy used to generate myeloid-specific Trem1 conditional knockout (*Lysm^Cre^Trem1^fl^*) mice.
(B) Experimental workflow showing preparation of lung single-cell suspensions, erythrocyte lysis, and neutrophil enrichment by magnetic bead separation, followed by downstream analyses including targeted energy metabolism, metabolic flow, oxidative respiratory chain assays, bulk RNA sequencing, and ATAC sequencing.
(C) Representative flow cytometry plots showing the purity of neutrophils isolated from peripheral blood and lung tissue of *Trem1^fl^* and *Lysm^Cre^Trem1^fl^* mice following ischemia-reperfusion. Neutrophils were identified as CD45⁺CD11b⁺Ly6G⁺ cells, with Trem1 expression assessed in parallel.


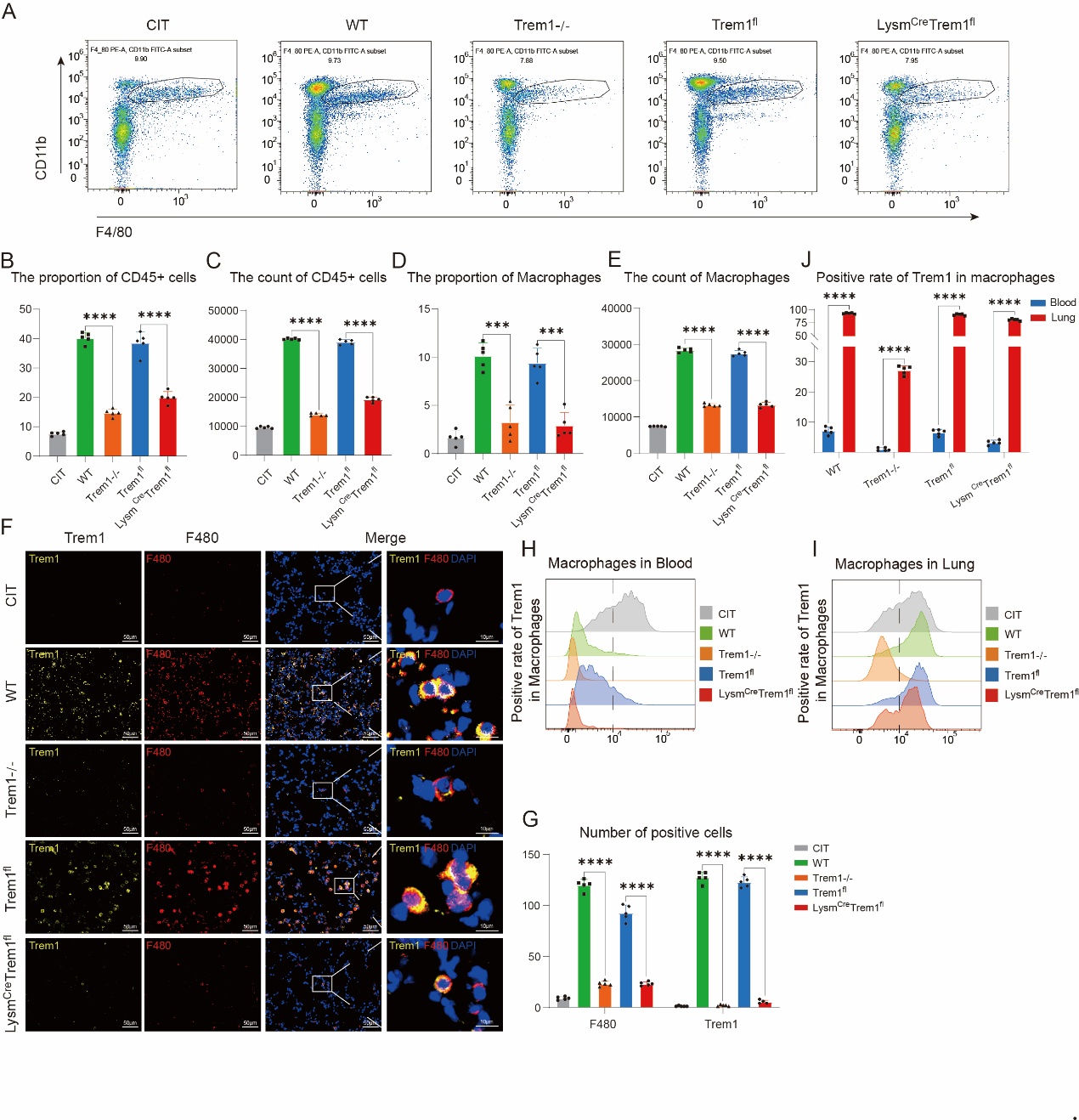


**Figure S9. Trem1 deficiency suppresses macrophage recruitment following lung transplantation. Related to Figure 3.**

(A) Representative flow cytometry plots showing the proportion of F4/80⁺ macrophages among CD45⁺ cells in lung grafts from indicated groups.
(B, C) Quantification of CD45⁺ immune cell counts and percentages (n = 5 per group).
(D, E) Quantification of macrophage counts and percentages in lung grafts (n = 5 per group).
(F) Representative immunofluorescence staining of Trem1 (yellow) and F4/80 (red) in lung grafts, with DAPI (blue) counterstain.
(G) Quantification of F4/80⁺ and Trem1⁺ cells from immunofluorescence images (n = 5 per group).
(H, I) Flow cytometry showing the frequency of Trem1⁺ macrophages in blood (H) and lung (I) across groups.
(J) Quantification of Trem1⁺ macrophage rates in blood versus lung tissue (n = 5 per group).

Results are expressed as mean ± SEM. *P < 0.05, **P < 0.01, ***P < 0.001, ****P < 0.0001.


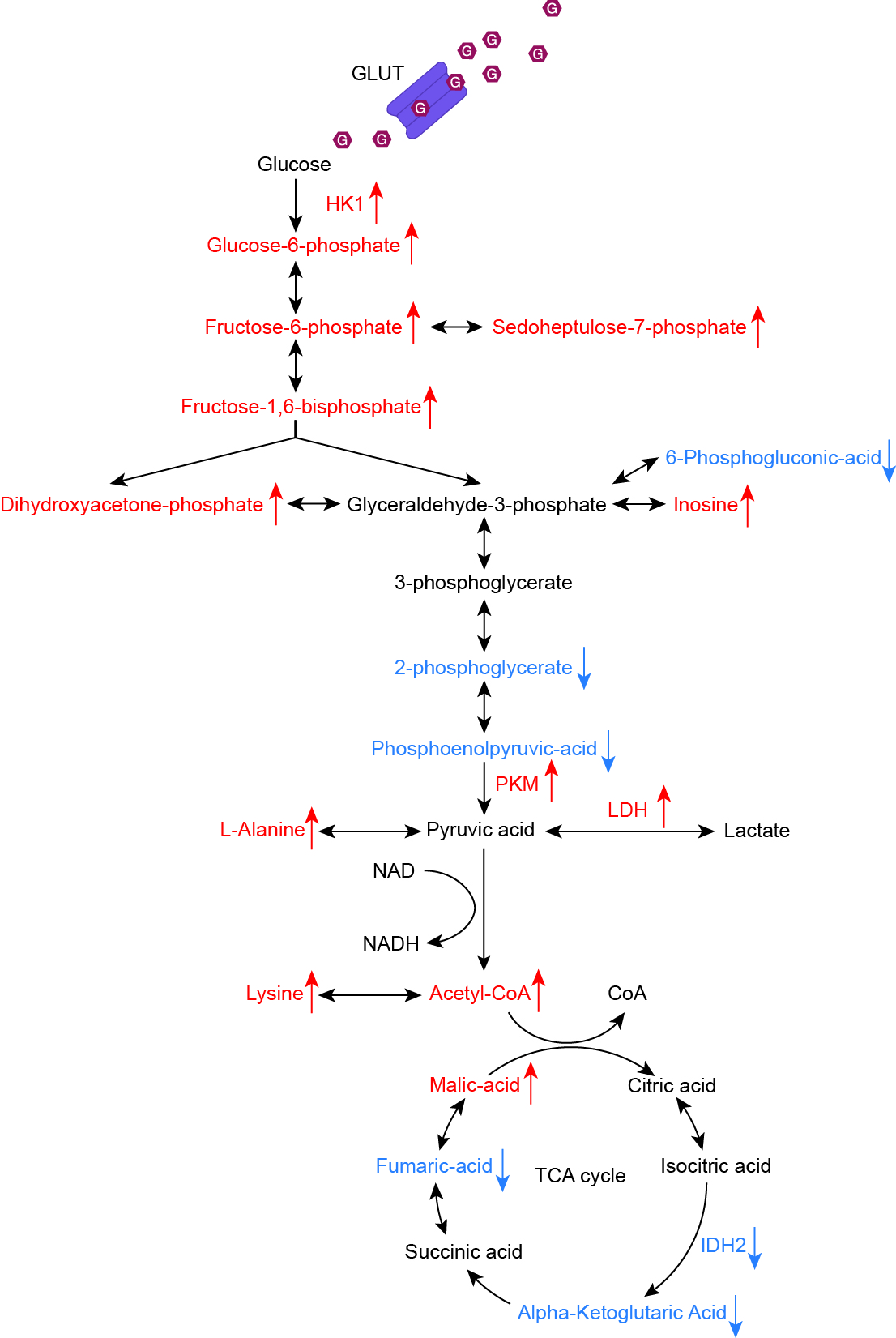


**Figure S10. Schematic representation of energy metabolism in neutrophils after lung transplantation and reperfusion, highlighting differentially regulated metabolites. Related to Figure 4.**


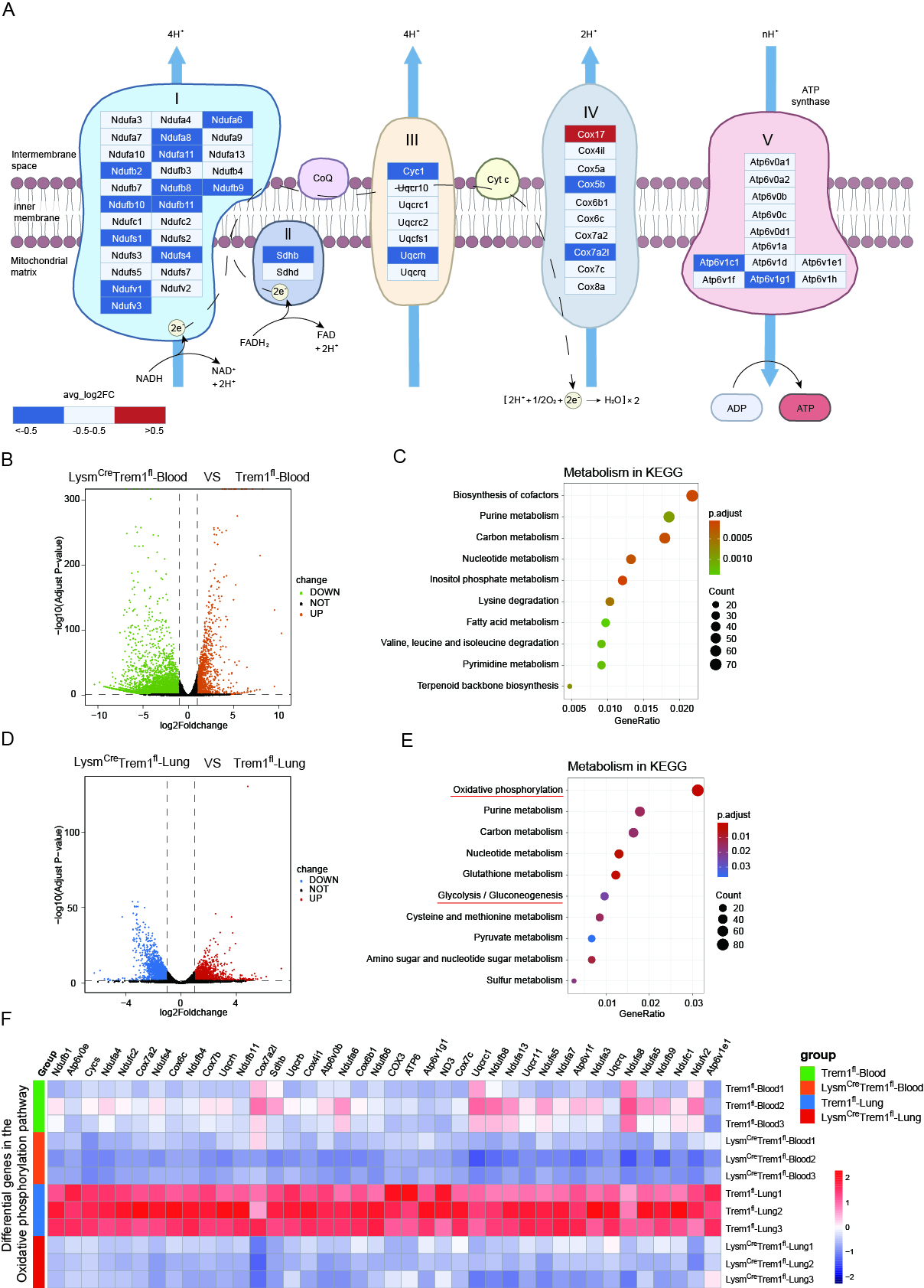


**Figure S11. Trem1 deletion suppresses oxidative phosphorylation in neutrophils. Related to Figure 4.**
(A) Differentially expressed genes related to oxidative phosphorylation in neutrophil subsets from WT and *Trem1-/-* mice by single-cell RNA sequencing.
(B) Volcano plot of differentially expressed genes in blood neutrophils from *Lysm^Cre^Trem1^fl^* versus *Trem1^fl^* mice.
(C) KEGG pathway enrichment of differentially expressed genes in blood neutrophils from *Lysm^Cre^Trem1^fl^* versus *Trem1^fl^* mice.
(D) Volcano plot of differentially expressed genes in lung neutrophils from *Lysm^Cre^Trem1^fl^* versus *Trem1^fl^* mice.
(E) KEGG pathway enrichment of differentially expressed genes in lung neutrophils from *Lysm^Cre^Trem1^fl^* versus *Trem1^fl^* mice.
(F) Heatmap of oxidative phosphorylation–related genes differentially expressed in blood and lung neutrophils from *Lysm^Cre^Trem1^fl^* and *Trem1^fl^* mice. Bulk RNA-seq analyses were performed with three biological replicates per group.


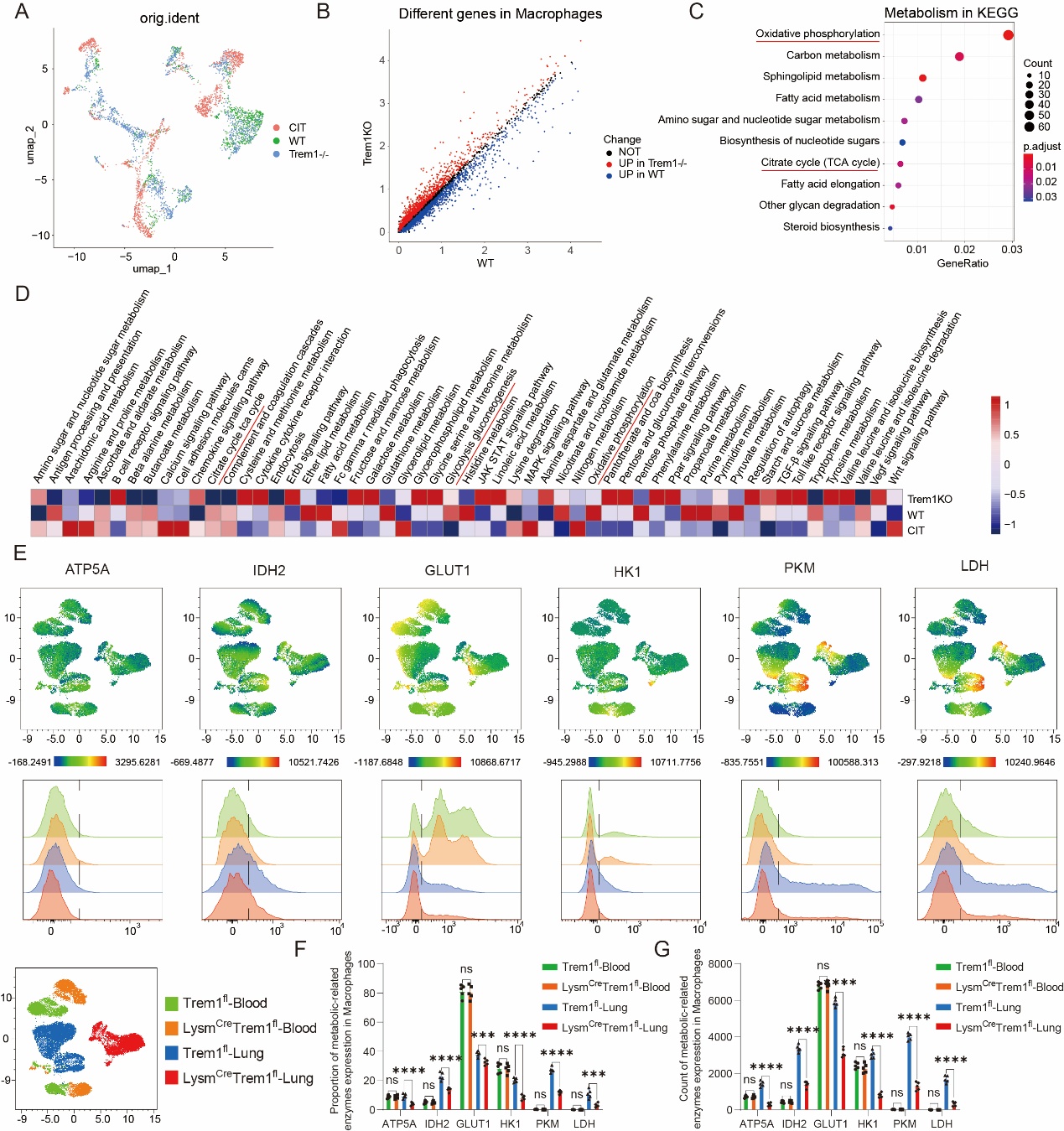


**Figure S12. Trem1 deficiency suppresses energy metabolism pathways in macrophages. Related to Figure 4.**

(A) UMAP projection of macrophage subsets from scRNA-seq data, colored by experimental group.
(B) Volcano plot of differentially expressed genes between macrophages isolated from lung grafts of *Trem1-/-* mice versus WT mice.
(C) Bubble plot of KEGG pathway enrichment analysis for differentially expressed genes in macrophages.
(D) Heatmap showing single-cell GSEA enrichment scores of hallmark metabolic pathways across macrophage subsets.
(E) Representative flow cytometry histograms depicting expression of key metabolic enzymes (related to oxidative phosphorylation, glycolysis, and TCA cycle) in macrophages.
(F, G) Quantification of the proportion (F) and absolute count (G) of macrophages positive for key metabolic enzymes across indicated groups.

Data are presented as mean ± SEM. Statistical significance was determined by one-way ANOVA with post hoc test. *P < 0.05, **P < 0.01, ***P < 0.001, ****P < 0.0001; ns, not significant.


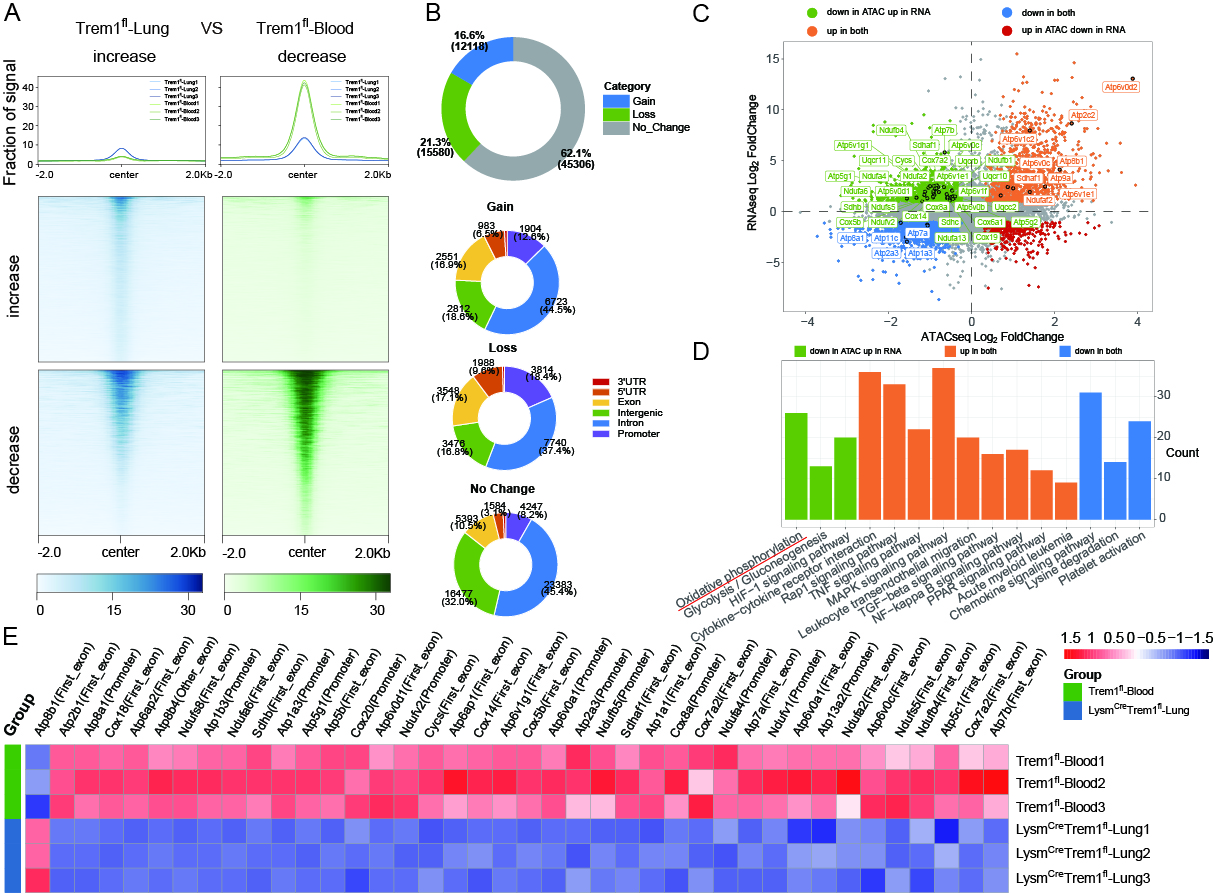


**Figure S13. Oxidative phosphorylation-related genes exhibit reduced chromatin accessibility in lung tissue neutrophils compared with resting blood neutrophils. Related to Figure 5.**

(A) Peak plots and heatmaps showing chromatin accessibility of neutrophils isolated from lung tissue versus resting blood neutrophils in *Trem1^fl^* mice (n = 3 mice per group).

(B) Quantification of differentially accessible chromatin regions between lung tissue neutrophils and resting blood neutrophils from *Trem1^fl^* mice (n = 3 mice per group).

(C) Four-quadrant volcano plot displaying genes with concordant differential expression and accessibility changes between lung tissue neutrophils and resting blood neutrophils from *Trem1^fl^* mice, as determined by integrated bulk RNA-seq and ATAC-seq analysis (n = 3 mice per group for each assay). Genes with P < 0.05 in both sequencing modalities are color-coded (green, blue, orange, and red).

(D) Pathway enrichment analysis of differentially expressed genes identified in the four-quadrant volcano plot.

(E) Genes associated with oxidative phosphorylation pathways showing differential chromatin accessibility between lung tissue neutrophils and resting blood neutrophils from *Trem1^fl^* mice.


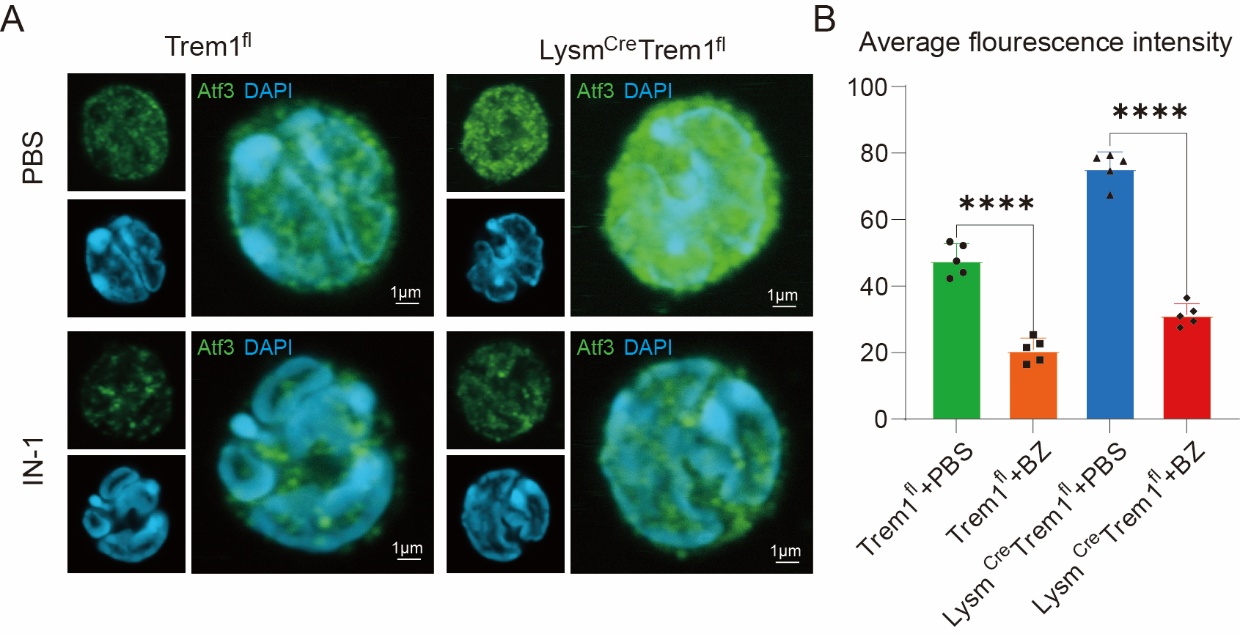


**Figure S14. IN-1 treatment reduces Atf3 expression in graft-infiltrating neutrophils. Related to Figure 6.**

(A) Representative confocal immunofluorescence images showing Atf3 expression in Ly6G⁺ neutrophils from lung grafts of *Trem1^fl^* and *Lysm^Cre^Trem1^fl^* mice treated with vehicle control or IN-1 (10 mg/kg, i.p.). Scale bars: 50 μm (overview), 10 μm (insets). (B) Quantification of Atf3 expression intensity in graft-infiltrating neutrophils across treatment groups (n = 5 mice per group).

Data are presented as mean ± SEM. Statistical significance was determined by one-way ANOVA with post hoc testing. *P < 0.05, **P < 0.01, ***P < 0.001, ****P < 0.0001.


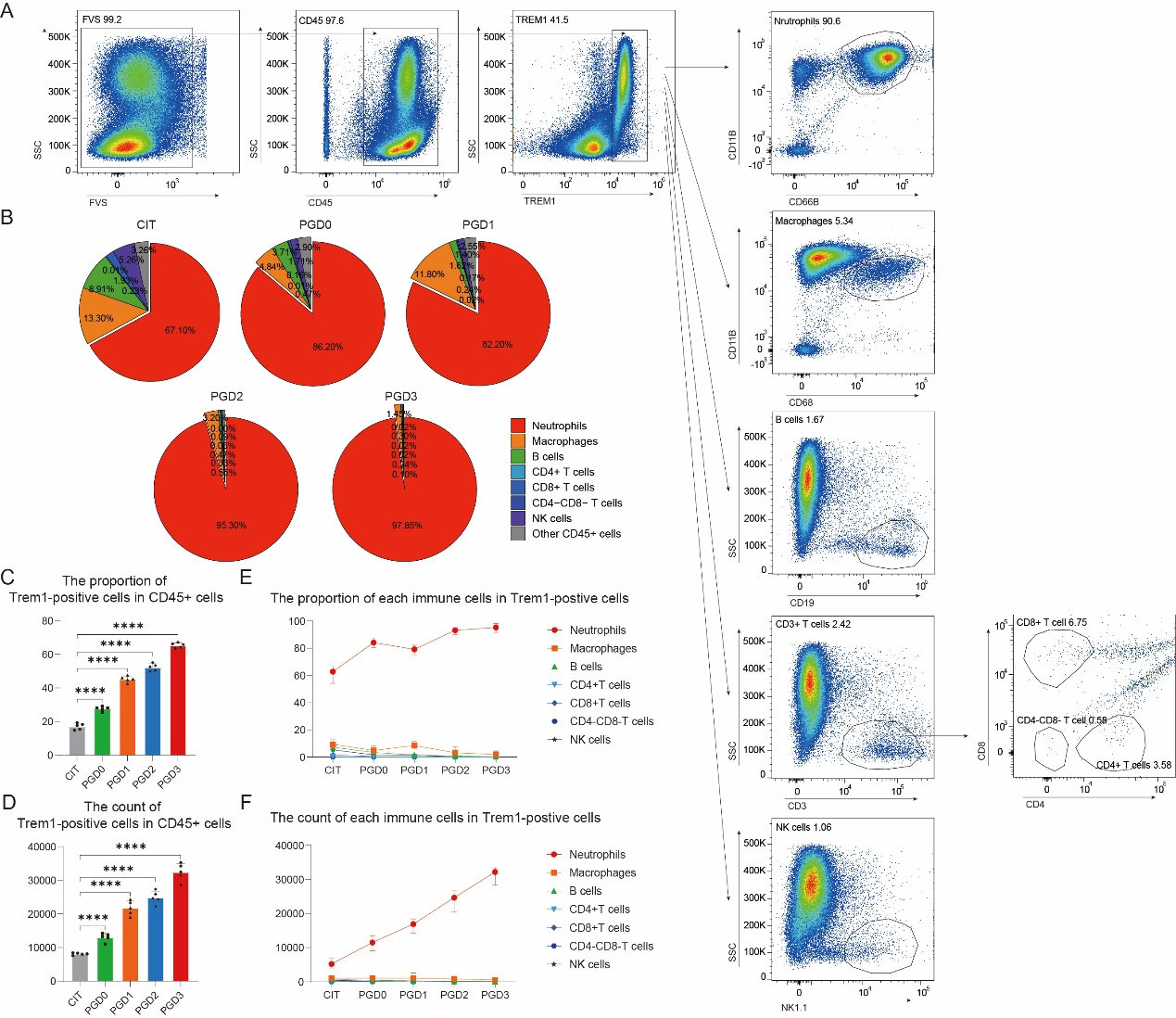


**Figure S15. TREM1-positive cells in human lung tissue are predominantly of myeloid origin, with neutrophils representing the major cellular population. Related to Figure 7.**

(A) Flow cytometry gating strategy used to identify TREM1⁺ immune cell populations in human lung tissues, including neutrophils (CD45⁺CD11b⁺CD66b⁺), macrophages (CD45⁺CD11b⁺CD68⁺), B cells (CD19⁺), CD4⁺ T cells, CD8⁺ T cells, NK cells (NK1.1⁺), and other CD45⁺ immune cells.

(B) Pie charts showing the relative composition of immune cell subsets among TREM1⁺ cells in human lung tissues across different pathological grades.

(C) Quantification of the absolute number of TREM1⁺ cells among total CD45⁺ immune cells in human lung tissues from the indicated groups.

(D) Quantification of the proportion of TREM1⁺ cells among total CD45⁺ immune cells in human lung tissues from the indicated groups.

(E) Quantification of the proportion of each immune cell subset within the TREM1⁺ cell population.

(F) Quantification of the absolute number of each immune cell subset within the TREM1⁺ cell population.

Data are presented as mean ± SEM. Statistical significance was determined by one-way ANOVA. **P < 0.01, ****P < 0.0001; ns, not significant.


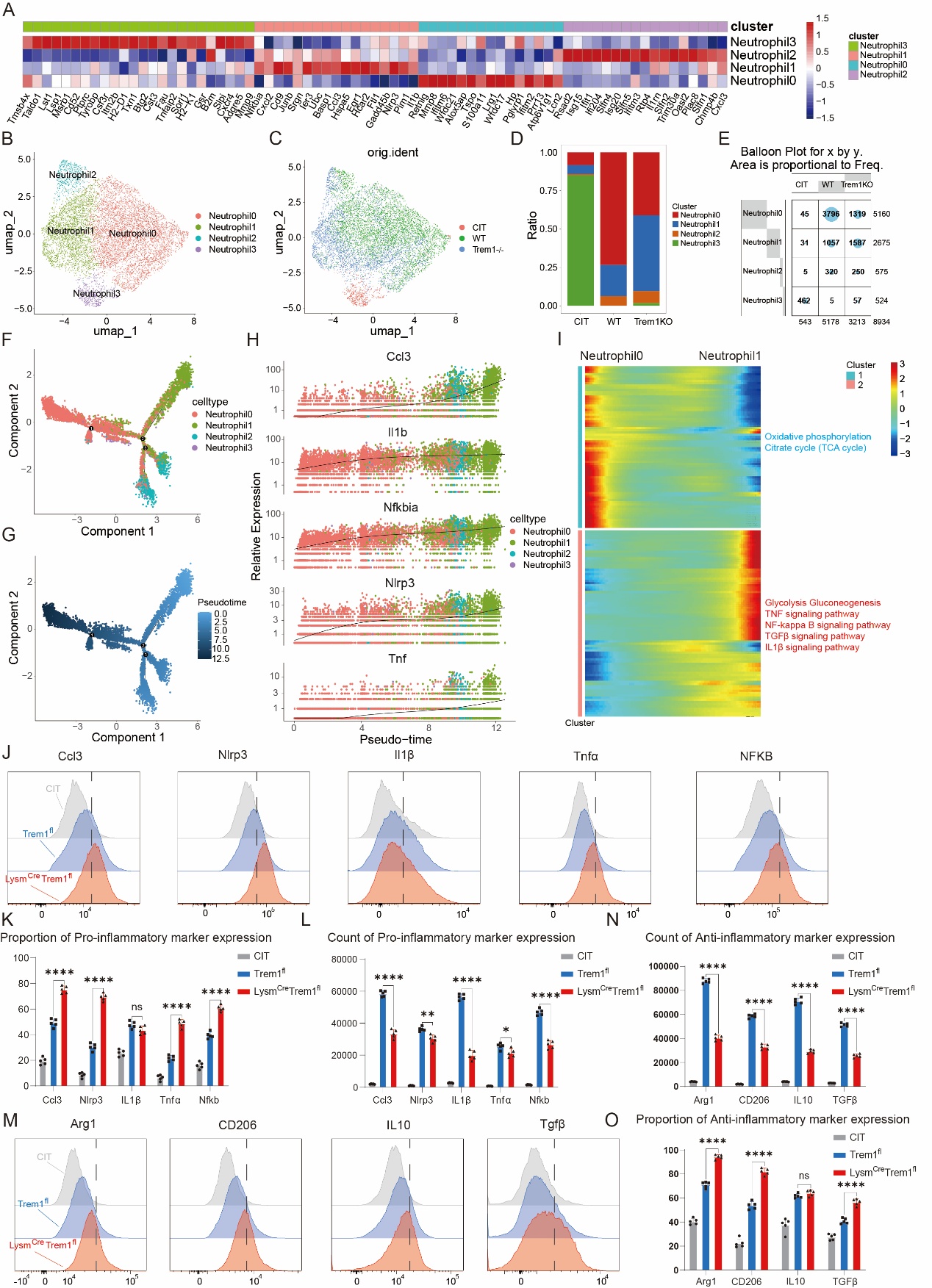


**Figure S16. Trem1 deletion promotes diverse neutrophil subtype differentiation patterns.**

(A) Heatmap showing marker genes defining neutrophil subpopulations identified by single-cell RNA sequencing.

(B) UMAP visualization of neutrophil subclusters across experimental groups.

(C) UMAP plots of neutrophils stratified by sample origin (CIT, WT, and *Trem1-/-* groups).

(D) Stacked bar chart showing relative proportions of neutrophil subpopulations across experimental groups.

(E) Bubble plot displaying absolute counts of neutrophil subpopulations from different sample sources.

(F) Pseudotime trajectory analysis of individual neutrophil clusters.

(G) Neutrophil pseudotime differentiation trajectory showing developmental progression.

(H) Expression dynamics of inflammatory genes along neutrophil pseudotime differentiation.

(I) Gene expression profiles of inflammatory pathway and energy metabolism pathway genes during neutrophil differentiation from cluster 0 to cluster 1.

(J) Representative flow cytometry plots showing expression of proinflammatory markers (Ccl3, Nlrp3, IL-1β, TNF-α, NF-κB) in lung tissue neutrophils across experimental groups.

(K, L) Quantification of neutrophils expressing proinflammatory genes (Ccl3, Nlrp3, IL-1β, TNF-α, NF-κB) by percentage (K) and absolute numbers (L).

(M) Representative flow cytometry plots showing expression of anti-inflammatory markers (Arg1, CD206, IL-10, TGF-β) in lung tissue neutrophils across experimental groups.

(N, O) Quantification of neutrophils expressing anti-inflammatory genes (Arg1, CD206, IL-10, TGF-β) by percentage (N) and absolute numbers (O).

Sample sizes: scRNA-seq analysis, n = 1 sample per group; flow cytometry validation, n = 5 mice per group. Data are presented as mean ± SEM. Statistical significance was determined by one-way ANOVA with post hoc testing. *P < 0.05, **P < 0.01, ***P < 0.001, ****P < 0.0001.


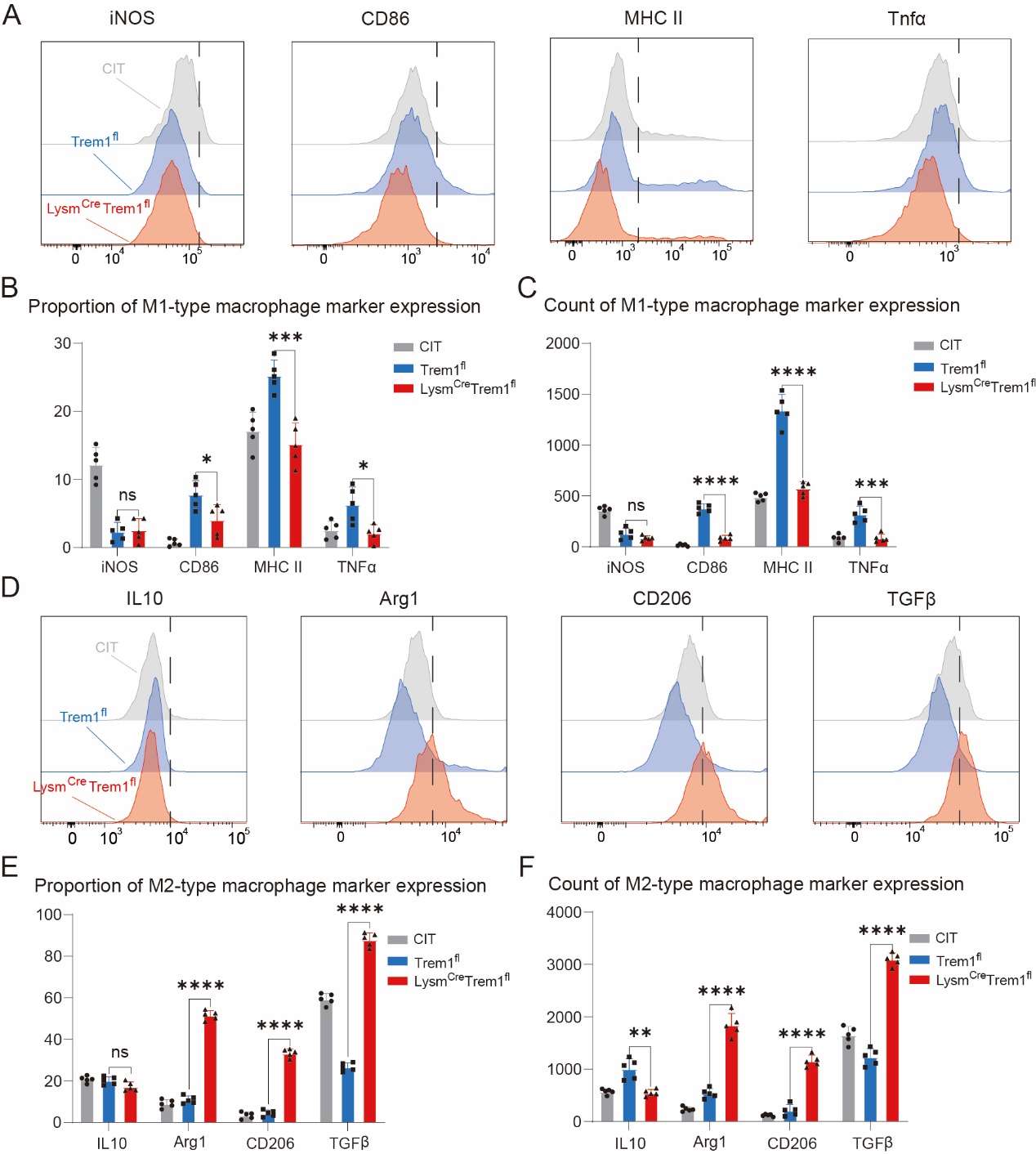


**Figure S17. Expression of pro-inflammatory and anti-inflammatory markers in macrophages.**

(A)UMAP visualization and histogram plots showing M1-type markers in macrophages detected by flow cytometry.

(B, C) Quantification of macrophages expressing M1-type markers by percentage (B) and absolute numbers (C) across experimental groups.

(D) UMAP visualization and histogram plots showing M2-type markers in macrophages detected by flow cytometry.

(E, F) Quantification of macrophages expressing M2-type markers by percentage (E) and absolute numbers (F) across experimental groups (n = 5 mice per group).

Data are presented as mean ± SEM. Statistical significance was determined by one-way ANOVA with post hoc testing. *P < 0.05, **P < 0.01, ***P < 0.001, ****P < 0.0001.
